# Integrated Physiological and Multi-Omics Analysis Reveals Selenium-Mediated Drought Tolerance in *Brassica napus* Seedlings

**DOI:** 10.3390/ijms27167232

**Published:** 2026-08-13

**Authors:** Shengyuan Gao, Mengjia Zhou, Zhongquan Jiang, Li Jia, Fanghua Zhu, Shi Chen, Ji Wang

**Affiliations:** 1 School of Geography and Planning, Chizhou University, Chizhou 247000, China; 2 State Key Laboratory of Crop Genetics & Germplasm Enhancement and Utilization, Nanjing Agricultural University, Nanjing 210095, China; 3 Research Center for Agricultural Ecological Resources and Environment, Chizhou University, Chizhou 247000, China; 4 Key Laboratory of Environmental Health Impact Assessment of Emerging Contaminants, Ministry of Ecology and Environment, School of Environmental Science and Engineering, Shanghai Jiao Tong University, Shanghai 200240, China; 5 East China Sea Fisheries Research Institute, Chinese Academy of Fishery Sciences, Shanghai 200090, China

**Keywords:** *Brassica napus*, selenium, drought stress, antioxidant enzymes, transcriptome, metabolome, integrated omics

## Abstract

Drought stress severely restricts early growth in *Brassica napus*, while selenium (Se) can enhance stress tolerance. This study examined how foliar sodium selenite pretreatment affects polyethylene glycol (PEG)-induced drought stress in *B. napus* seedlings. Seedlings were sprayed with 10, 20, or 40 mg L^−1^ Na_2_SeO_3_ for 7 days and then exposed to 10% (*w*/*v*) PEG 6000 for 2 days. Drought reduced fresh weight and increased malondialdehyde (MDA), O_2_·^−^ production, H_2_O_2_ accumulation, and antioxidant enzyme activities. The response was nonlinear, and 20 mg L^−1^ Na_2_SeO_3_ was most effective. Relative to drought alone, this treatment increased fresh weight from 2.6 to 5.1 g plant^−1^ and reduced MDA, O_2_·^−^, and H_2_O_2_ by 50.5%, 50.9%, and 44.3%, respectively. Notably, catalase (CAT) and superoxide dismutase (SOD) activities declined markedly from the drought-induced levels, indicating lower oxidative pressure rather than enhanced enzymatic scavenging. Integrated transcriptomic and metabolomic analyses showed associations between selenium pretreatment and pathways related to photosynthesis, carbon fixation, porphyrin metabolism, phenylpropanoid biosynthesis, and selenocompound metabolism. Together, these findings suggest that foliar application of 20 mg L^−1^ Na_2_SeO_3_ improves drought tolerance in *B. napus* seedlings by alleviating oxidative pressure rather than simply increasing antioxidant enzyme activity.

## 1. Introduction

Episodes of soil water deficit are projected to increase in frequency, duration, severity and spatial extent under ongoing climate change [1,2]. This trend poses a major challenge to temperate oilseed production, including oilseed rape (*Brassica napus* L.). In major production regions such as Europe and China’s Yangtze River basin, drought at the seedling and pre-flowering stages has been reported to cause substantial yield losses, commonly 10–30% and exceeding 50% under severe conditions [3,4]. The seedling stage is particularly sensitive to water deficit because early leaf expansion, photosynthetic establishment and metabolic homeostasis influence subsequent biomass accumulation and yield formation [5]. Drought responses in the field are often further complicated by concurrent heat, high irradiance and nutrient limitation, which together intensify oxidative and metabolic stress in plant cells [6].

At the cellular level, drought stress involves not only water limitation but also oxidative imbalance. Stomatal closure and perturbation of chloroplast function can cause excessive reduction in the photosynthetic electron transport chain, promoting electron leakage and energy transfer reactions that generate reactive oxygen species (ROS), including superoxide radical (O_2_·^−^), singlet oxygen (^1^O_2_), hydrogen peroxide (H_2_O_2_) and hydroxyl radical (•OH) [7]. Although ROS at controlled levels act as important signals in plant development and stress responses, excessive ROS accumulation causes oxidative damage to lipids, proteins and nucleic acids, thereby disrupting cellular homeostasis and restricting growth [8]. Maintaining ROS homeostasis therefore depends both on limiting ROS production and on scavenging by enzymatic antioxidants such as superoxide dismutase (SOD), catalase (CAT), peroxidase (POD) and ascorbate peroxidase (APX), together with non-enzymatic antioxidants [9]. SOD dismutates O_2_·^−^ into H_2_O_2_ and O_2_, CAT decomposes H_2_O_2_ mainly in peroxisomes, whereas POD and APX participate in H_2_O_2_ scavenging in the cytosol, apoplast and organelles. When ROS production exceeds the capacity of this antioxidant network, ROS accumulation is usually accompanied by increased injury markers such as malondialdehyde (MDA) and protein carbonyls [10].

Selenium occupies a distinctive position in plant stress physiology. Although Se is not generally considered essential for higher plants, Se supplied within a beneficial range has been reported to improve plant tolerance to salinity, heat, heavy metal toxicity and drought, whereas concentrations above this range can be phytotoxic [11,12,13,14,15]. Proposed mechanisms include ROS scavenging by Se-containing compounds or Se-induced redox metabolites, regulation of glutathione and other thiol pools, modulation of stomatal behaviour, and changes in the expression of genes associated with hormone signalling and antioxidant defence [16,17,18]. Nevertheless, the mechanistic basis of Se-mediated drought tolerance remains incompletely understood. Previous studies have often emphasized antioxidant enzyme activities, while growth responses and biochemical changes have not always been evaluated together. In addition, antioxidant responses are not always interpreted together with transcriptomic or metabolomic evidence, making it difficult to place these changes within a broader regulatory framework. Moreover, Se responses vary with plant species, genotype, Se form, application method, and stress conditions [19,20]. This variability highlights the need to evaluate a range of Se concentrations alongside growth, biochemical, and molecular responses within a unified experimental framework.

The response of plants to Se is strongly concentration-dependent. As a broad tissue-based reference, beneficial effects on plant growth and stress resistance are often reported at approximately 1–10 mg Se kg^−1^ dry weight [21]. However, phytotoxic effects may occur at tissue concentrations as low as a few mg Se kg^−1^ dry weight in sensitive species, whereas other non-accumulator plants may tolerate concentrations approaching 100 mg Se kg^−1^ dry weight [16,22]. Based on Se accumulation under natural seleniferous conditions, plants are generally classified as non-accumulators (<100 mg Se kg^−1^ dry weight), Se accumulators (100–1000 mg Se kg^−1^ dry weight), and Se hyperaccumulators (>1000 mg Se kg^−1^ dry weight) [21,22,23]. These categories refer to tissue Se accumulation and tolerance and are distinct from the external Se doses applied through soil, nutrient solution, or foliar spraying. For the solution-based studies discussed below, reported concentrations are additionally expressed as elemental Se equivalents in mg Se L^−1^ to aid comparison.

Dose-dependent effects of Se on biomass production and antioxidant enzyme activities have been reported across plant systems. In hydroponically grown apple seedlings, biomass, net photosynthetic rate, and antioxidant enzyme activities peaked when Se was supplied through the nutrient solution at 9 μmol L^−1^ (0.71 mg Se L^−1^) and declined sharply at concentrations of 24 μmol L^−1^ or higher (≥1.90 mg Se L^−1^) [19]. In hydroponically grown *B. napus*, supplying 25 μmol L^−1^ Se(IV) (1.97 mg Se L^−1^) as sodium selenite through the nutrient solution promoted growth, photosynthetic performance, and redox balance, whereas 50 and 100 μmol L^−1^ Se(IV) (3.95 and 7.90 mg Se L^−1^, respectively) progressively impaired oxidative metabolism and reduced biomass, with cultivar-dependent sensitivity [24]. Under PEG-induced drought, Se nanoparticle suspensions at 2 and 5 mg L^−1^ were applied to the perlite substrate and improved canola growth, whereas antioxidant enzyme responses differed between the two concentrations [25].

*B. napus* has an appreciable capacity for Se enrichment and biotransformation, converting part of the absorbed inorganic Se into organic Se species. In Se-enriched rapeseed seedlings and flowering stalks, selenocystine, methylselenocysteine, selenomethionine, selenite, and selenate were detected. Organic Se accounted for 64.18–90.20% of the detected Se in seedlings and 94.38–98.47% in flowering stalks [26]. Foliar Se application can also increase Se concentrations in different rapeseed organs. A field study showed that foliar application of sodium selenate at 25 and 50 g Se ha^−1^ caused stepwise increases in Se concentrations in all examined organs, with concentrations generally following the order leaves > stems > roots > siliques ≈ seeds [27]. Se accumulation may subsequently affect the quality of mature rapeseed products. Following oil extraction, more than 99% of the Se in seeds was retained in the rapeseed meal, whereas less than 1% was found in the oil. Se application was also associated with lower erucic acid and higher oleic acid contents [28]. Together, these findings support the potential of Se enrichment for the biofortification of rapeseed products, particularly rapeseed meal. However, the quantitative accumulation data from the foliar field study were obtained with sodium selenate, while the present study applied sodium selenite to seedlings and did not quantify tissue Se concentration or speciation. The present results therefore address drought responses at the seedling stage and do not permit assessment of the nutritional quality or dietary safety of mature rapeseed products.

Despite growing knowledge of Se accumulation and biotransformation in *B. napus*, its role in drought tolerance remains insufficiently understood. Physiological and biochemical responses to Se under water deficit have been examined in rice and wheat [29,30]. In *B. napus*, a recent study investigated Se nanoparticles under drought [25], while other seedling studies focused on salinity or arsenic stress using a limited set of physiological and antioxidant indices [31,32]. Studies at the crop scale have mainly evaluated agronomic performance and yield under drought [14]. Taken together, current evidence does not fully explain how concentration-dependent Se effects on growth and oxidative status are related to broader molecular responses in *B. napus* under drought. Integrated analyses linking physiological and biochemical responses with transcriptomic and metabolomic changes therefore remain needed.

In this study, *B. napus* seedlings received foliar pretreatment with 10, 20, or 40 mg L^−1^ Na_2_SeO_3_, equivalent to 4.57, 9.13, and 18.26 mg Se L^−1^, respectively, for 7 d before exposure to 10% (*w*/*v*) PEG 6000 for 2 d. The experimental design was guided by studies using Se pretreatment before PEG exposure in rapeseed seedlings [33], and by foliar Na_2_SeO_3_ studies evaluating 5–40 mg L^−1^ under drought conditions in other crops [34]. The three concentrations were arranged in twofold increments to examine responses across the tested range. Physiological and biochemical responses were first compared among treatments, and the treatment showing the strongest overall protection was selected for subsequent transcriptomic and metabolomic analyses. By integrating these datasets, this study aimed to characterise the physiological and molecular responses associated with sodium selenite pretreatment under drought stress in *B. napus* seedlings.

## 2. Results

### 2.1. Selenium Pretreatment Mitigated Drought-Induced Growth Inhibition

**Figure 1:**
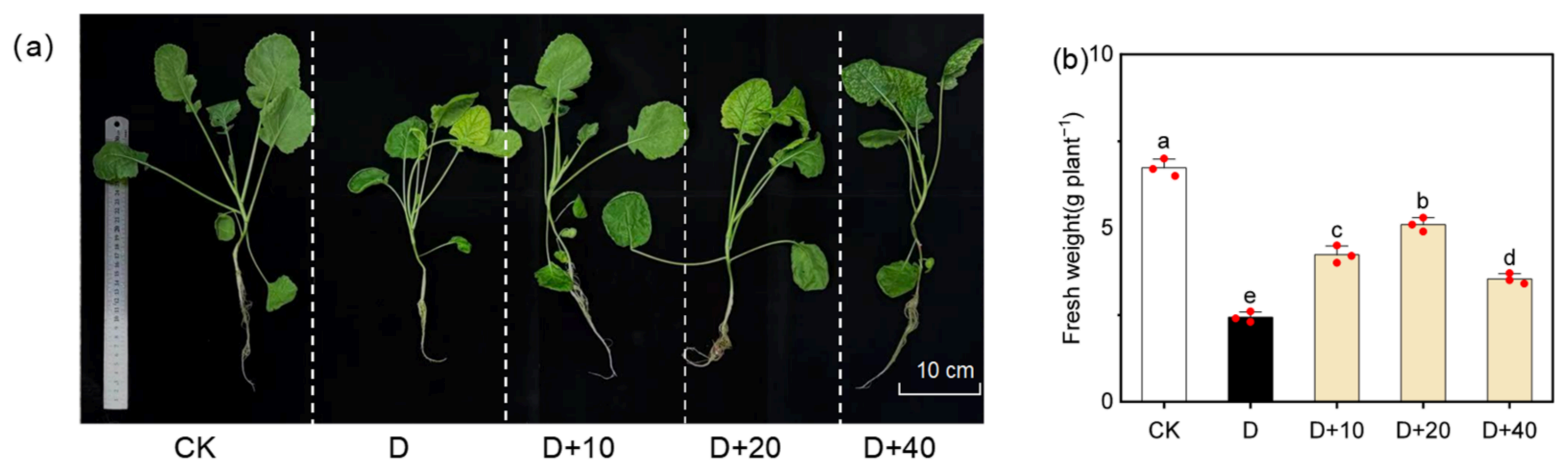
Effect of selenium on rapeseed growth under drought stress. (**a**) Representative seedling phenotypes of the five treatments after 2 d of drought stress. CK, well-watered control; D, 10% (*w*/*v*) PEG-induced drought; D+10, D+20 and D+40, 10% (*w*/*v*) PEG combined with foliar Na_2_SeO_3_ pretreatment at 10, 20 and 40 mg L^−1^ for 7 d, respectively. Scale bar = 10 cm. (**b**) Fresh weight per plant (mean ± SD, *n* = 3 biological replicates). Different letters indicate significant differences at *p* < 0.05 (Duncan’s multiple range test).

Two days of 10% (*w*/*v*) PEG 6000 treatment markedly reduced seedling growth. Compared with CK, drought-treated plants showed visible wilting and smaller shoots (Figure 1a). Foliar Se pretreatment for 7 d before drought partially alleviated these phenotypic changes. Among the Se-pretreated groups, D+20 plants maintained the best shoot development, with more expanded leaves and a larger plant size than D+10 and D+40. D+10 plants also showed milder wilting than the drought group, whereas D+40 plants remained smaller than D+20 plants.

Fresh weight (FW) per plant followed the same trend (Figure 1b). The mean fresh weight decreased from 6.7 g plant^−1^ in CK to 2.6 g plant^−1^ under drought, corresponding to a 61.2% reduction. Foliar Se pretreatment partially restored FW, with values of 4.2, 5.1 and 3.5 g plant^−1^ in D+10, D+20 and D+40, respectively. D+20 produced the highest fresh weight among the drought-treated groups. In contrast, D+40 was significantly lower than both D+10 and D+20. These results indicate that Se alleviated drought-induced growth inhibition in a concentration-dependent manner, with 20 mg L^−1^ Na_2_SeO_3_ being the most effective concentration under the present conditions.

### 2.2. Selenium Reduced Oxidative Damage and Moderated Antioxidant Enzyme Activity

**Figure 2:**
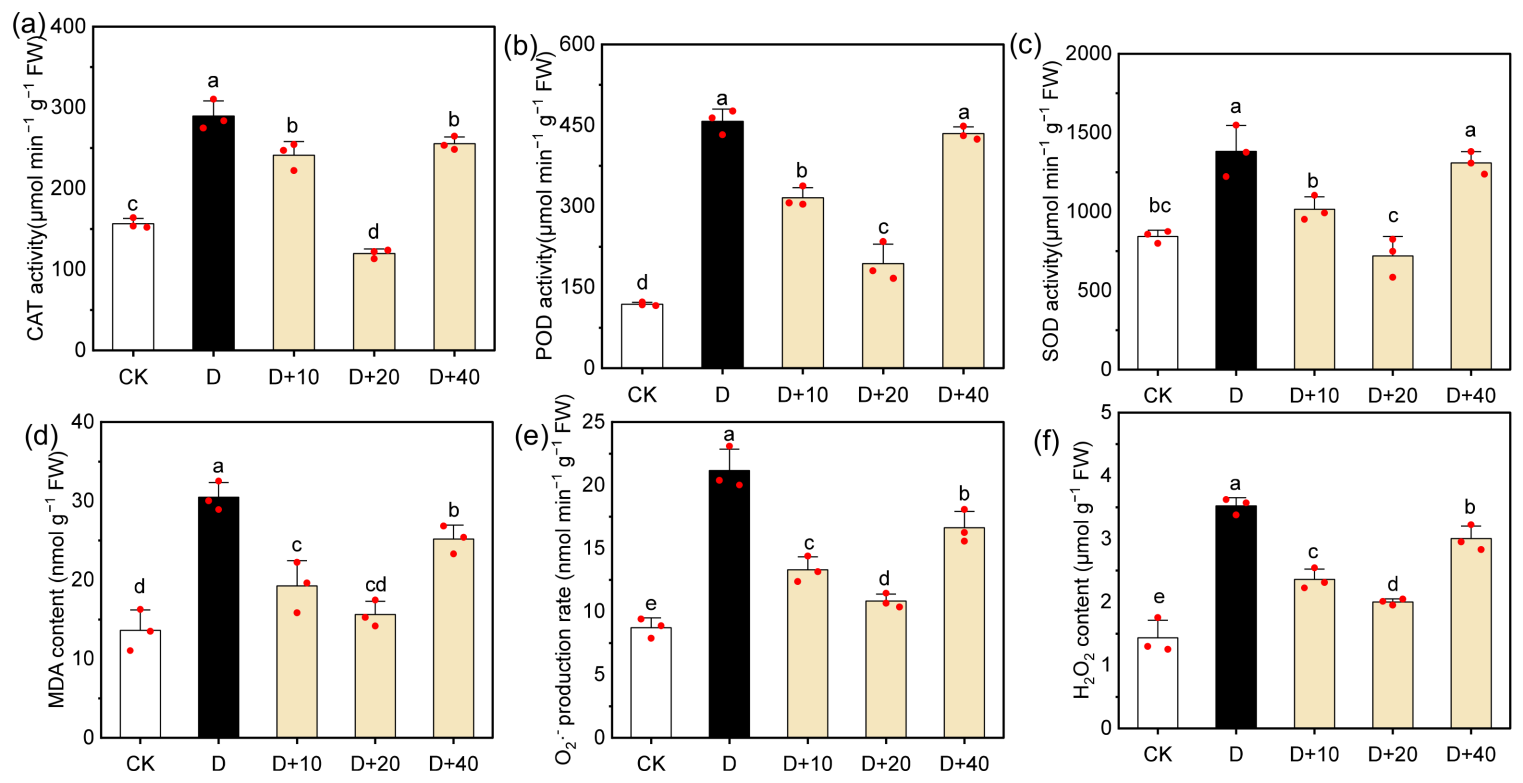
Effects of selenium pretreatment on antioxidant enzyme activities, lipid peroxidation, and oxidative stress indicators in drought-stressed rapeseed seedlings. (**a**–**c**) Activities of CAT, POD, and SOD, respectively; (**d**) MDA content, an oxidative damage marker; (**e**) O_2_·^−^ production rate and (**f**) H_2_O_2_ content, indicators of oxidative stress. Treatments are as described in Figure 1. Bars represent the mean ± SD (*n* = 3 biological replicates). Different letters indicate significant differences at *p* < 0.05.

Drought stress markedly disturbed redox status in rapeseed seedlings (Figure 2). CAT, POD and SOD activities in D were 1.7, 4.0 and 1.6 times those in CK, respectively (Figure 2a–c). MDA content, O_2_·^−^ production rate and H_2_O_2_ content also increased under drought, reaching 30.9 nmol g^−1^ FW, 21.8 nmol min^−1^ g^−1^ FW and 3.5 μmol g^−1^ FW, respectively. Each value was more than twice that in CK (Figure 2d–f).

Se pretreatment reduced oxidative damage under drought, and the extent varied with Se concentration. Among the drought groups, D+20 had the lowest MDA content, O_2_·^−^ production rate and H_2_O_2_ content, with values of 15.3 nmol g^−1^ FW, 10.7 nmol min^−1^ g^−1^ FW and 1.95 μmol g^−1^ FW, respectively. Relative to D, these values decreased by 50.5%, 50.9% and 44.3%. D+10 also lowered these injury markers, but less strongly than D+20. D+40 retained higher MDA, O_2_·^−^ and H_2_O_2_ levels than D+20. Antioxidant enzyme activities followed a similar pattern. In D+20, CAT and SOD activities declined to values similar to or lower than those in CK, whereas POD activity remained above CK but was clearly lower than in D (Figure 2a–c). D+10 showed intermediate values, and D+40 retained relatively high enzyme activities. Thus, 20 mg L^−1^ Na_2_SeO_3_ gave the clearest reduction in oxidative damage and lowered the enzyme activation caused by drought. Based on these results, D+20 was selected for subsequent transcriptome and metabolome analyses.

### 2.3. Overview of Differentially Expressed Genes Under Drought and Selenium Pretreatment

**Figure 3:**
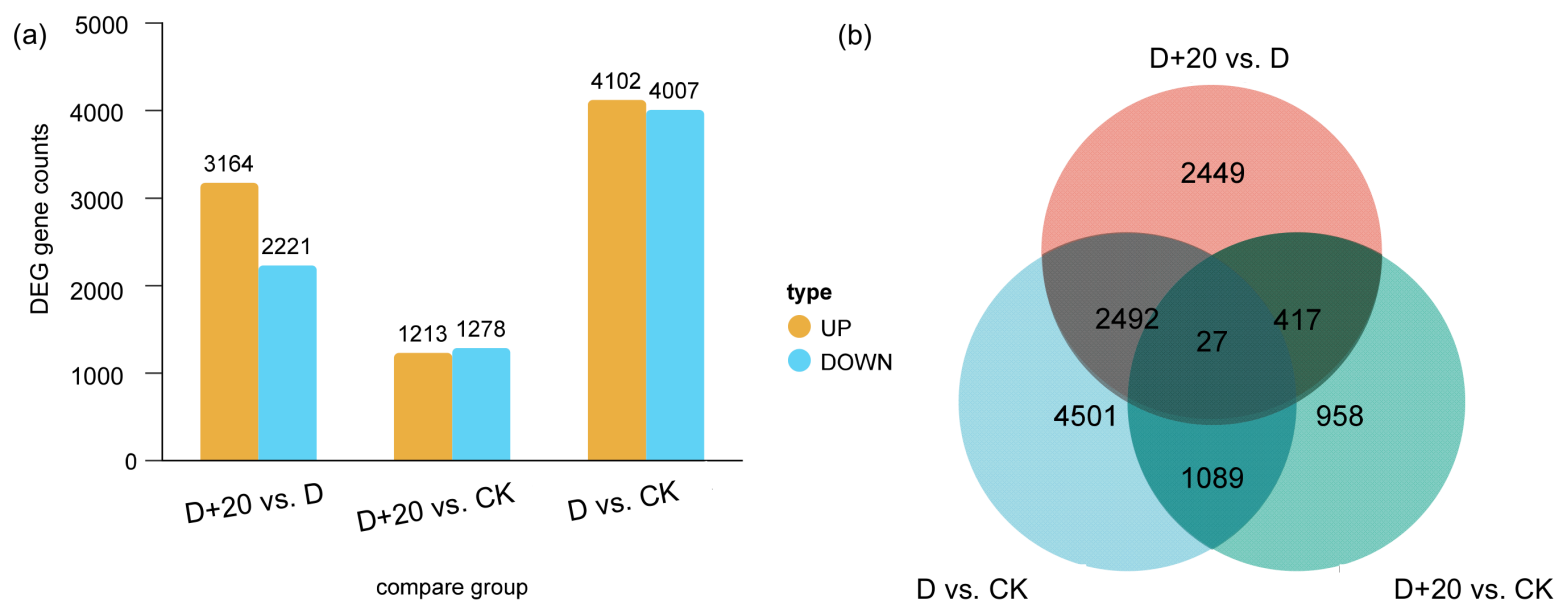
Overview of differentially expressed genes (DEGs) in rapeseed seedlings. (**a**) Number of up- and down-regulated DEGs in the three pairwise comparisons, defined using |log_2_FC| ≥ 1 and *p* value < 0.05. (**b**) Venn diagram of DEGs among the three comparisons. CK, well-watered control; D, drought without selenium; D+20, drought plus foliar Na_2_SeO_3_ at 20 mg L^−1^.

RNA-seq was performed on CK-, D- and D+20-treated seedlings. Each library produced more than 6 Gb of clean data after fastp filtering, with Q30 above 93% and mapping rates above 95% to the ZS11 reference genome. To validate the RNA-seq results, six representative Se-responsive genes were examined by qRT-PCR. Their expression patterns were broadly consistent with the RNA-seq trends, supporting the reliability of the transcriptome data (Figure S1; Table S1). A total of 8109 differentially expressed genes (DEGs) were identified between D and CK (4102 upregulated and 4007 downregulated). The D+20 vs. CK comparison yielded 2491 DEGs (1213 upregulated and 1278 downregulated), and the D+20 vs. D comparison yielded 5385 DEGs (3164 upregulated and 2221 downregulated) (Figure 3a, Table S2). Venn analysis showed that 4501 DEGs were specific to the D vs. CK comparison, 958 were specific to D+20 vs. CK, and 2449 were specific to D+20 vs. D (Figure 3b). Only 27 DEGs were shared among all three comparisons, indicating that drought stress and Se pretreatment induced largely distinct transcriptional responses in rapeseed seedlings.

### 2.4. Functional Enrichment of Differentially Expressed Genes

**Figure 4:**
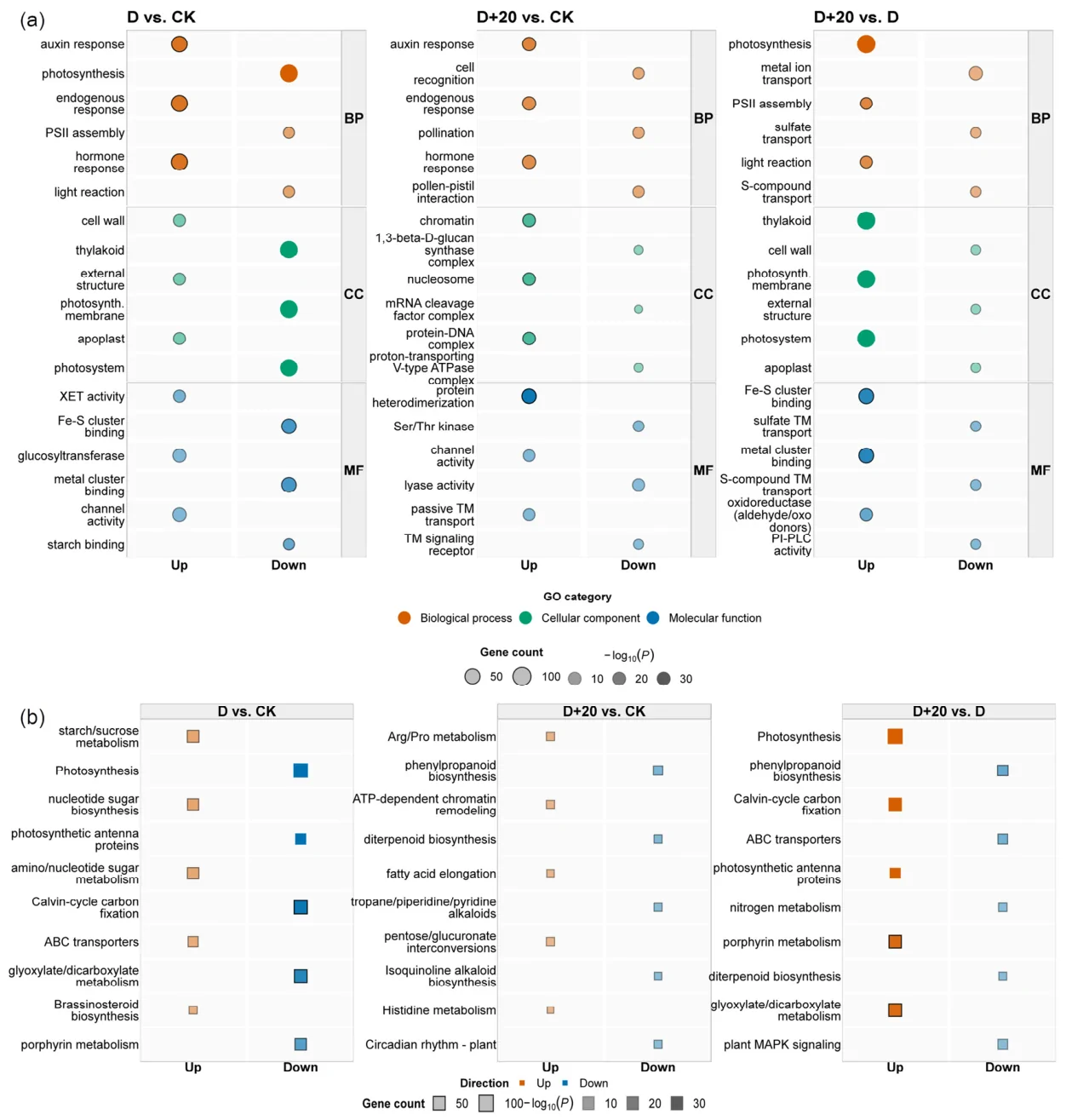
Functional enrichment analysis of DEGs in rapeseed seedlings. (**a**) For each comparison and regulation direction, the three GO terms with the smallest raw *p* values were selected within biological process (BP), cellular component (CC) and molecular function (MF). (**b**) For each comparison and regulation direction, the five KEGG pathways with the smallest raw *p* values were selected. Circles represent GO terms, and squares represent KEGG pathways. Symbol size is proportional to the number of mapped DEGs; therefore, smaller squares in (**b**) indicate KEGG pathways containing fewer mapped DEGs. Symbol opacity indicates −log_10_(*raw p*), while colour denotes the GO category in (**a**) and the regulation direction in (**b**). Full results are provided in Tables S3 and S4. CK, well-watered control; D, drought treatment without selenium; D+20, drought following foliar pretreatment with 20 mg L^−1^ Na_2_SeO_3_.

Gene Ontology (GO) enrichment was conducted separately for the upregulated and downregulated DEGs in each comparison (Figure 4a, Table S3). Figure 4a summarizes the three terms with the smallest raw *p* values within each GO category and regulation direction. In D vs. CK, the top-ranked terms for upregulated genes were related mainly to auxin, endogenous stimulus and hormone responses, together with cell wall and apoplast-associated components. For downregulated genes, the top-ranked terms were dominated by photosynthesis-related processes and structures, including photosynthesis, photosystem II assembly, the light reaction, thylakoid, photosynthetic membrane and photosystem. Iron-sulfur cluster and metal cluster binding were also represented among the leading molecular function terms. In D+20 vs. CK, upregulated genes were associated mainly with hormone responses, chromatin, nucleosome and protein-DNA complexes, as well as protein heterodimerization. Downregulated genes were associated with cell recognition, pollination and pollen–pistil interaction, together with several protein-complex, transporter and signaling functions. In D+20 vs. D, photosynthesis-related processes, thylakoid-associated structures and metal-cluster binding were concentrated among the top-ranked terms for upregulated genes. The downregulated genes were associated mainly with metal, sulfate and sulfur-compound transport and with cell wall and apoplast-associated components.

Kyoto Encyclopedia of Genes and Genomes (KEGG) enrichment was likewise analysed separately for upregulated and downregulated genes (Figure 4b, Table S4). In D vs. CK, the top-ranked pathways for upregulated genes included starch and sucrose metabolism, biosynthesis of nucleotide sugars, amino sugar and nucleotide sugar metabolism, ABC transporters and brassinosteroid biosynthesis. The downregulated genes were associated mainly with photosynthesis, photosynthetic antenna proteins, carbon fixation by the Calvin cycle, glyoxylate and dicarboxylate metabolism and porphyrin metabolism. In D+20 vs. CK, upregulated genes were associated with arginine and proline metabolism, ATP-dependent chromatin remodeling, fatty acid elongation, pentose and glucuronate interconversions and histidine metabolism. The downregulated genes were associated mainly with phenylpropanoid, diterpenoid and alkaloid biosynthesis and plant circadian rhythm. In D+20 vs. D, photosynthesis, carbon fixation, photosynthetic antenna proteins, porphyrin metabolism and glyoxylate and dicarboxylate metabolism ranked highest among upregulated genes, whereas phenylpropanoid biosynthesis, ABC transporters, nitrogen metabolism, diterpenoid biosynthesis and plant MAPK signaling ranked highest among downregulated genes. Overall, photosynthesis-related functions were prominent among genes downregulated in D vs. CK and among genes upregulated in D+20 vs. D.

### 2.5. Metabolite Profiling and Analysis of Differentially Accumulated Metabolite Features

An untargeted LC-MS/MS metabolomics approach was used to characterise treatment-related changes at the metabolite-feature level. Metabolite names were assigned through database matching and should be regarded as putative annotations because they were not confirmed using authentic standards. Accordingly, the metabolomics data were interpreted in terms of relative feature abundance, chemical-class composition and pathway-level patterns. In the multi-omics integration, metabolite features were used to examine covariance with gene expression rather than to establish validated metabolite identities, causal regulation or confirmed gene–metabolite relationships. After quality filtering, 4008 metabolite features were retained for downstream analysis. The Class I, Class II, and Class III annotations in Table S5 were assigned using the structure-based ChemOnt taxonomy implemented in ClassyFire and represent progressively more specific levels of chemical classification. Class I denotes the broadest chemical category, whereas Class II and Class III provide increasingly specific subcategories. These fields are distinct from the HMDB-derived SuperClass, Class, and SubClass annotations and from the KEGG identifiers and pathway mappings reported separately in Table S5. At the Class I level, the overall feature profile was dominated by lipids and lipid-like molecules (27.70%), followed by organoheterocyclic compounds (15.89%), organic acids and derivatives (15.53%), organic oxygen compounds (12.81%), phenylpropanoids and polyketides (11.45%), and benzenoids (10.38%). Together, these six classes accounted for 93.76% of the overall putatively annotated feature profile.

**Figure 5:**
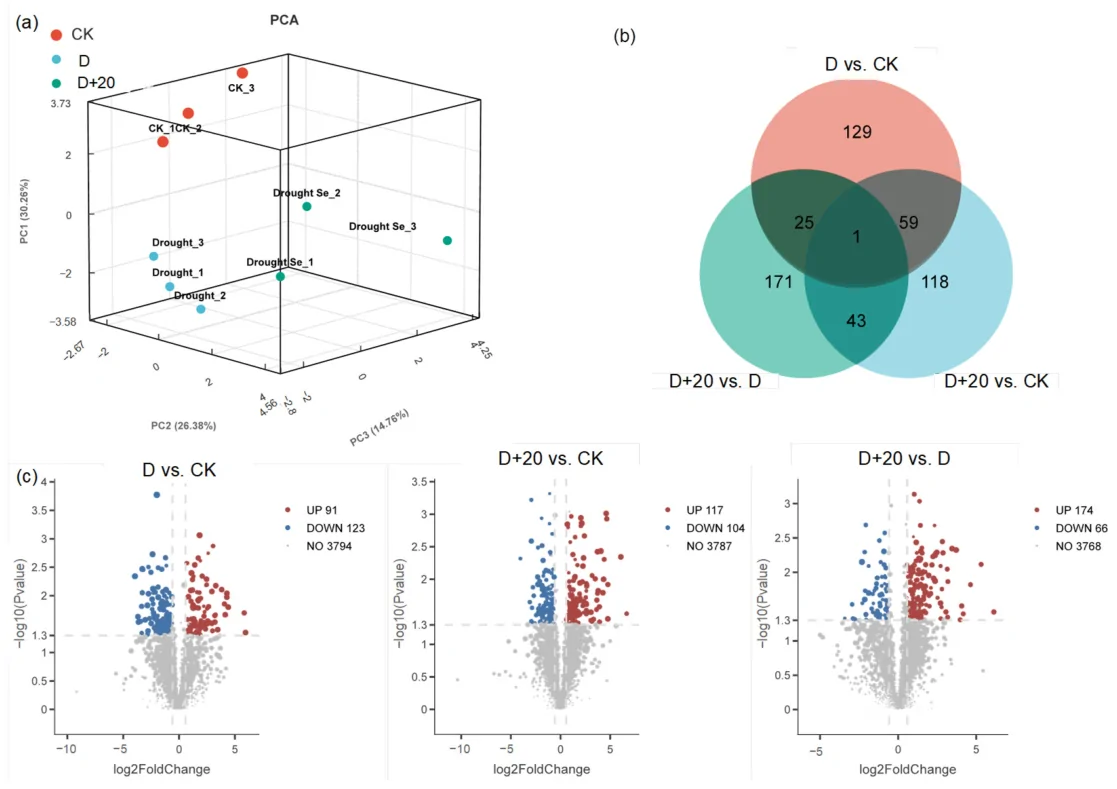
Metabolomic profiling of rapeseed seedlings. (**a**) Principal component analysis (PCA) of the three treatment groups. (**b**) Venn diagram of candidate differentially accumulated metabolites (DAMs) in the three pairwise comparisons. (**c**) Volcano plots of candidate DAMs in D vs. CK, D+20 vs. CK and D+20 vs. D. The two vertical dashed lines indicate the log_2_FC cutoffs at FC = 0.67 and FC = 1.5, whereas the horizontal dashed line indicates raw *p* = 0.05. Red and blue points represent increased and decreased candidate DAMs, respectively, meeting VIP ≥ 1.0, raw *p* < 0.05 and the specified FC thresholds; the remaining features are grey. CK, well-watered control; D, drought without selenium; D+20, drought plus foliar Na_2_SeO_3_ at 20 mg L^−1^.

Principal component analysis (PCA) and orthogonal partial least-squares discriminant analysis (OPLS-DA) were then used to assess sample distribution and identify treatment-responsive metabolite features. In the PCA based on the full metabolite-feature matrix, CK, D and D+20 showed distinct distributions in the three-dimensional space defined by PC1, PC2 and PC3, which together explained 71.4% of the total variance (Figure 5a). Replicates generally grouped by treatment, although some within-group dispersion was evident. The pooled quality control (QC) samples clustered closely in the QC-inclusive PCA, supporting the analytical stability of the liquid chromatography-tandem mass spectrometry (LC–MS/MS) dataset (Figure S2). Candidate differentially accumulated metabolite features (DAMs) were screened using variable importance in projection (VIP) ≥ 1.0, raw *p* < 0.05, and either fold change (FC) > 1.5 or FC < 0.67. Benjamini–Hochberg correction was applied separately to the raw *p* values of all 4008 retained features in each pairwise comparison. No individual feature had a BH-adjusted *p* value (FDR) < 0.05 in D vs. CK, D+20 vs. CK, or D+20 vs. D. Accordingly, the features meeting the combined VIP, fold-change, and raw-*p*-value criteria were treated as candidate, putatively annotated metabolite features suitable for exploratory chemical-class, pathway, and covariance analyses, rather than as FDR-supported differentially abundant features, confirmed metabolite identities, or validated biomarkers. Under these exploratory criteria, 214 candidate DAMs were identified in D vs. CK, including 91 increased and 123 decreased features. The D+20 vs. CK comparison contained 221 candidate DAMs, with 117 increased and 104 decreased features, whereas the D+20 vs. D comparison contained 240 candidate DAMs, with 174 increased and 66 decreased features (Figure 5c, Table S5). Venn analysis showed that 129 DAMs were specific to D vs. CK, 171 to D+20 vs. D and 118 to D+20 vs. CK. Only one DAM was shared by all three comparisons (Figure 5b). Thus, D vs. CK contained more decreased than increased candidate features, whereas D+20 vs. D showed the opposite pattern. The Class I composition of the DAMs was broadly similar across the three comparisons, although the proportions of the major classes differed. The six largest classes together accounted for 89.1–92.5% of the DAMs in each comparison. In D vs. CK, lipids and lipid-like molecules were predominant (72 features, 33.6%), followed by organoheterocyclic compounds (33, 15.4%), organic acids and derivatives (31, 14.5%), and phenylpropanoids and polyketides (27, 12.6%). In D+20 vs. CK, lipids and lipid-like molecules (59, 26.7%) and organoheterocyclic compounds (45, 20.4%) were the two largest classes. In D+20 vs. D, organoheterocyclic compounds (54, 22.5%), lipids and lipid-like molecules (52, 21.7%), and organic acids and derivatives (51, 21.3%) were similarly represented. Thus, the treatment-responsive features were distributed across several major chemical classes rather than being restricted to a single class (Table S5).

### 2.6. KEGG Enrichment Analysis of Differentially Accumulated Metabolites

**Figure 6:**
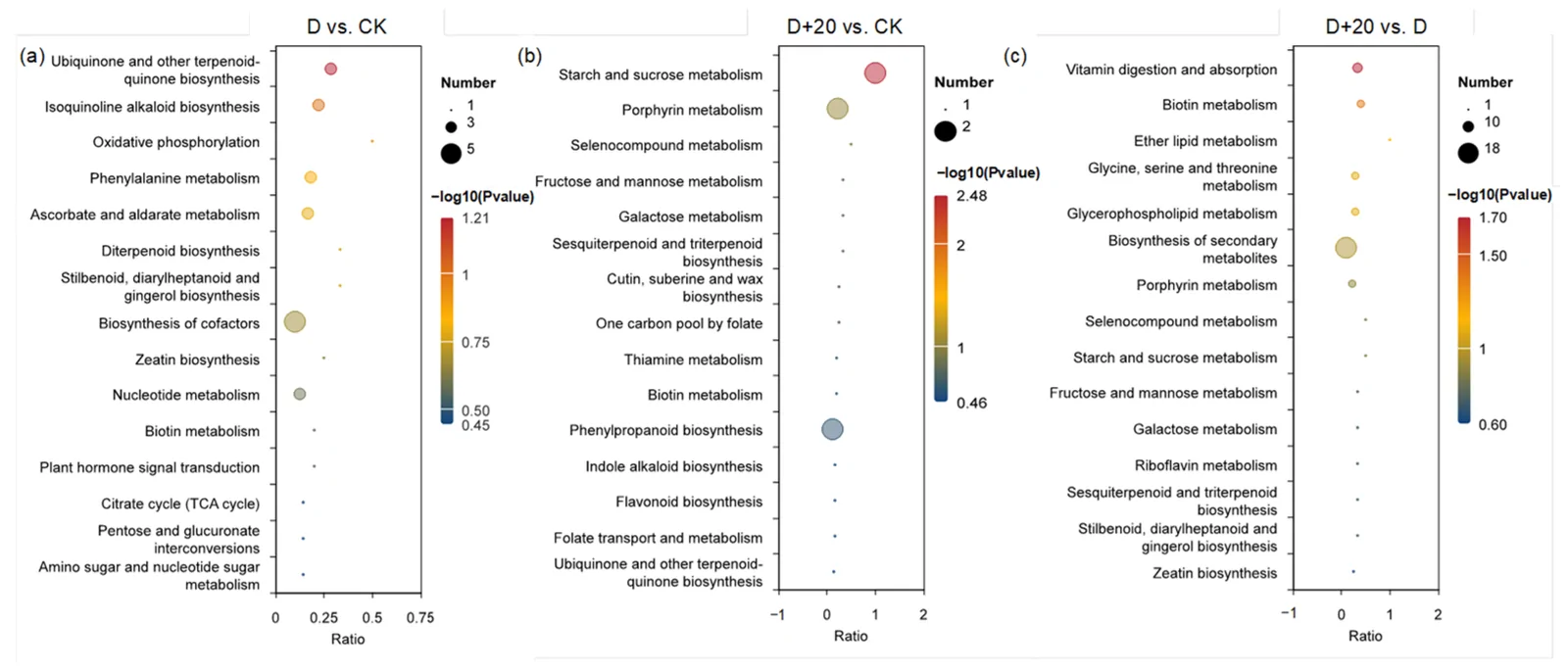
KEGG pathway enrichment analysis of DAMs in rapeseed seedlings. (**a**) KEGG pathway enrichment results for the D vs. CK comparison; (**b**) KEGG pathway enrichment results for the D+20 vs. CK comparison; (**c**) KEGG pathway enrichment results for the D+20 vs. D comparison. For visualization, the 15 pathways with the smallest raw pathway-enrichment *p* values among those containing at least one mapped candidate DAM are shown. The x axis indicates the enrichment ratio. Bubble size represents the number of DAMs mapped to each pathway, and bubble color represents −log_10_(raw *p* value). Candidate DAMs were screened using VIP ≥ 1.0, FC > 1.5 or FC < 0.67, and raw *p* < 0.05. CK, well-watered control; D, drought without selenium; D+20, drought plus foliar Na_2_SeO_3_ at 20 mg L^−1^.

KEGG pathway enrichment analysis of the DAMs in the three pairwise comparisons is shown in Figure 6 and Table S6. In D vs. CK, the top-ranked pathways mainly included ubiquinone and other terpenoid-quinone biosynthesis, isoquinoline alkaloid biosynthesis, oxidative phosphorylation, phenylalanine metabolism, and ascorbate and aldarate metabolism (Figure 6a). In D+20 vs. CK, the top-ranked pathways included starch and sucrose metabolism, porphyrin metabolism, selenocompound metabolism, fructose and mannose metabolism, and galactose metabolism (Figure 6b). In D+20 vs. D, the top-ranked pathways mainly involved vitamin digestion and absorption, biotin metabolism, ether lipid metabolism, glycine, serine and threonine metabolism, glycerophospholipid metabolism, porphyrin metabolism, and selenocompound metabolism (Figure 6c). Porphyrin metabolism and selenocompound metabolism appeared among the top-ranked pathways in both comparisons involving D+20, whereas ascorbate and aldarate metabolism appeared among the top-ranked pathways only in D vs. CK.

Several DAMs with putative annotations contributed to these top-ranked KEGG pathway patterns (Tables S5 and S6). In D vs. CK, features annotated as hydroxyphenyllactic acid and 3-polyprenyl-4,5-dihydroxybenzoate decreased and mapped to ubiquinone and other terpenoid-quinone biosynthesis. Phenylacetaldehyde and phenylacetylglutamine also decreased and mapped to phenylalanine metabolism, whereas threonic acid increased and mapped to ascorbate and aldarate metabolism. In D+20 vs. CK, trehalose and D-fructose decreased and mapped to starch and sucrose metabolism, whereas pheophorbide A and pheophytin A increased and mapped to porphyrin metabolism. Se-methylselenocysteine also increased and mapped to selenocompound metabolism. In D+20 vs. D, riboflavin and nicotinamide increased, while biotinyl-5′-AMP and biocytin increased and mapped to biotin metabolism. The feature annotated as sn-glycero-3-phosphocholine decreased and mapped to ether lipid and glycerophospholipid metabolism. In contrast, protochlorophyllide and pheophytin A increased and mapped to porphyrin metabolism, and Se-methylselenocysteine again increased and mapped to selenocompound metabolism. Several additional features with sulfur-containing annotations, including S-methylglutathione, γ-glutamyl-S-allylcysteine, methylcysteine, and 5-methylthio-D-ribose, increased, whereas N-acetylmethionine decreased. Together, the comparisons involving D+20 highlighted changes in carbohydrate-, lipid-, porphyrin-, biotin-, and selenocompound-related pathway mappings, together with several sulfur-containing metabolite features. Other pathway-mapped DAMs are provided in Tables S5 and S6. All compound names represent putative annotations and should therefore be interpreted at the metabolite-feature level rather than as confirmed metabolite identities.

### 2.7. Integrated KEGG Pathway Analysis of DEGs and DAMs

**Figure 7:**
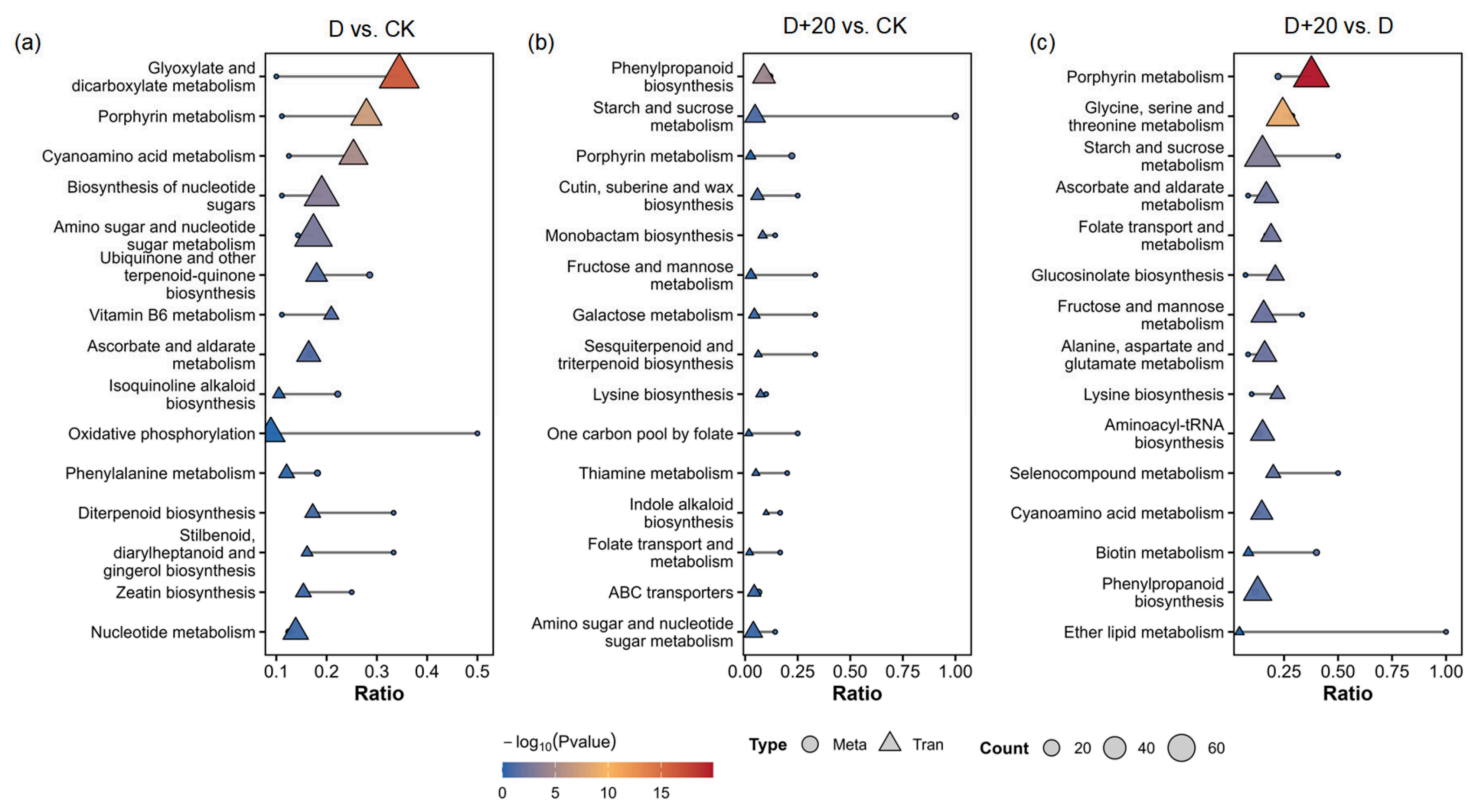
Integrated KEGG enrichment analysis of transcriptome and metabolome data in rapeseed seedlings. (**a**) D vs. CK, (**b**) D+20 vs. CK and (**c**) D+20 vs. D. Triangles represent transcriptome enrichment based on differentially expressed genes (DEGs, Tran), and circles represent metabolome enrichment based on differentially accumulated metabolites (DAMs, Meta). For each pathway, horizontal lines connect the transcriptome and metabolome signals when both are present. The x axis indicates the enrichment ratio. Symbol size represents the number of mapped DEGs or DAMs, and symbol color represents −log_10_ (*p* value) for the corresponding enrichment result. DEGs were identified using |log_2_FC| ≥ 1 and *p* < 0.05; Candidate DAMs were screened using VIP ≥ 1.0, FC > 1.5 or FC < 0.67, and raw *p* < 0.05. CK, well-watered control; D, drought without selenium; D+20, drought plus foliar Na_2_SeO_3_ at 20 mg L^−1^.

To identify pathways represented at both the gene and metabolite levels, KEGG results for DEGs and DAMs were compared in each of the three pairwise comparisons (Figure 7). Pathways that contained mapped features in both datasets were examined further, and the full list is provided in Table S7. In many cases, the statistical signal was stronger at the transcriptome level, whereas the metabolite level was represented by a smaller number of mapped DAMs.

In D vs. CK, glyoxylate and dicarboxylate metabolism showed the strongest signal at the gene level and also contained a corresponding DAM. Other pathways that contained both DEGs and DAMs included porphyrin metabolism, biosynthesis of nucleotide sugars, amino sugar and nucleotide sugar metabolism, ascorbate and aldarate metabolism, and stilbenoid, diarylheptanoid and gingerol biosynthesis (Figure 7a). In D+20 vs. CK, phenylpropanoid biosynthesis showed the strongest signal at the gene level, whereas starch and sucrose metabolism showed the strongest signal at the metabolite level. Both pathways contained DEGs and DAMs, as did porphyrin metabolism, sesquiterpenoid and triterpenoid biosynthesis, and cutin, suberine and wax biosynthesis (Figure 7b). In D+20 vs. D, porphyrin metabolism showed the strongest signal at the gene level, followed by glycine, serine and threonine metabolism, and both pathways also contained corresponding DAMs. Other pathways represented at both levels included starch and sucrose metabolism, selenocompound metabolism, phenylpropanoid biosynthesis, stilbenoid, diarylheptanoid and gingerol biosynthesis, and ascorbate and aldarate metabolism (Figure 7c).

Across the three comparisons, porphyrin metabolism contained both DEGs and DAMs in all of them. Phenylpropanoid biosynthesis showed this pattern in the two comparisons involving D+20. Stilbenoid, diarylheptanoid and gingerol biosynthesis, together with ascorbate and aldarate metabolism, appeared at both levels in D vs. CK and D+20 vs. D. Selenocompound metabolism appeared at both levels only in D+20 vs. D.

### 2.8. O2PLS Integration of Transcriptome and Metabolome Data

**Figure 8:**
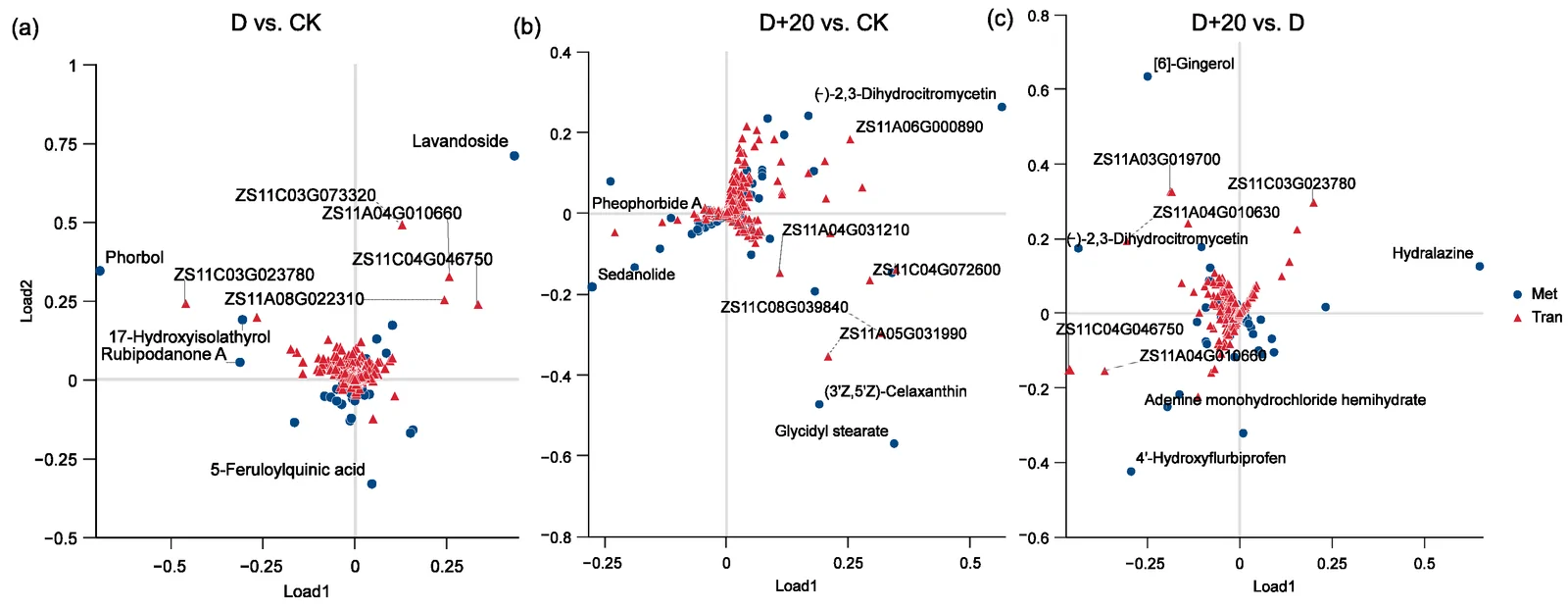
O2PLS loading plots integrating transcriptome and metabolome data in rapeseed seedlings. (**a**) D vs. CK, (**b**) D+20 vs. CK and (**c**) D+20 vs. D. Red triangles represent genes, and blue circles represent metabolite features detected by untargeted LC-MS/MS metabolomics. Labels indicate variables with high loading values in each comparison. Metabolite names are putative annotations and should be interpreted at the feature level. CK, well-watered control; D, drought without selenium; D+20, drought plus foliar Na_2_SeO_3_ at 20 mg L^−1^.

To complement the pathway-level integration described above, O2PLS was used to examine covariance between gene expression and LC-MS/MS metabolite-feature abundance (Figure 8). This analysis does not depend on pathway annotation and was used to identify genes and metabolite features that contributed strongly to the joint variation between the transcriptome and metabolome datasets. In the loading plots, points farther from the origin had larger loading values and therefore contributed more strongly to the covariance structure.

In D vs. CK, high-loading metabolite features had tentative database annotations including lavandoside, phorbol, 17-hydroxyisolathyrol, rubipodanone A and 5-feruloylquinic acid (Figure 8a). In D+20 vs. CK, high-loading features were tentatively annotated as (-)-2,3-dihydrocitromycetin, sedanolide, (3′Z,5′Z)-celaxanthin and glycidyl stearate; genes with large loadings included *ZS11A06G000890*, *ZS11A04G031210*, *ZS11C04G072600*, *ZS11C08G039840* and *ZS11A05G031990* (Figure 8b). In D+20 vs. D, high-loading metabolite features included features tentatively annotated as [6]-gingerol-like and (-)-2,3-dihydrocitromycetin-like, together with two features whose best database matches were hydralazine-like and 4′-hydroxyflurbiprofen-like (Figure 8c). Because the latter two annotations are not typical endogenous plant metabolites and were not confirmed using authentic standards, they were retained only as feature-level markers and were not used for compound-specific biological interpretation.

Overall, the O2PLS results support treatment-dependent covariance between gene expression and metabolite features. However, given the putative nature of the untargeted metabolite annotations, these results were used only to describe covariance patterns between the two omics layers, not to infer validated metabolite identities, causal regulation or confirmed gene–metabolite relationships.

## 3. Discussion

### 3.1. The Effect of Se Under Drought Depends on Treatment Concentration

Foliar Se alleviated growth inhibition caused by drought in *B. napus* seedlings, but the response was not linear. The protective effect was strongest at 20 mg L^−1^ Na_2_SeO_3_ (9.13 mg Se L^−1^) and declined at 40 mg L^−1^ Na_2_SeO_3_ (18.26 mg Se L^−1^), although D+40 remained beneficial relative to drought alone (Figure 1). This pattern is consistent with a hormetic-type dose response, in which the benefit peaks at an intermediate Se supply and diminishes as the dose increases [35]. Similar patterns have been reported in other crops. In apple seedlings grown hydroponically with Se concentrations from 0 to 48 µmol L^−1^ (0–3.79 mg Se L^−1^), biomass, net photosynthetic rate and antioxidant enzyme activity peaked at 9 µmol L^−1^ (0.71 mg Se L^−1^) and declined sharply at concentrations equal to or above 24 µmol L^−1^ (≥1.90 mg Se L^−1^) [19]. In cowpea, 15 to 30 µg kg^−1^ Se in soil increased seed number and yield, whereas 45 µg kg^−1^ reduced the benefit. These soil-mass-based application rates are not directly comparable with solution concentrations. In hydroponic cowpea, root flavonoid levels peaked at 5 µmol L^−1^ (0.39 mg Se L^−1^) and declined at higher concentrations [36]. Similarly, in tobacco subjected to graded soil-moisture conditions, two foliar applications of sodium selenite at 15 mg L^−1^ (6.85 mg Se L^−1^) produced the strongest growth and biomass responses, whereas 20 mg L^−1^ (9.13 mg Se L^−1^) reduced biomass relative to the corresponding untreated control under all three moisture regimes [34].

The effective Se concentrations reported for *B. napus* are not directly comparable because nominal treatment concentrations do not represent equivalent plant exposure. In a field study, a 7.06 μmol L^−1^ Se foliar treatment (0.56 mg Se L^−1^), supplied as sodium selenite, was initiated at the 10 d seedling stage and repeated at 25 d intervals, either alone or combined with seed priming [14]. In another experiment, 2 or 5 mg L^−1^ Se nanoparticles were applied to the perlite root zone every other day for 3 weeks [25]. These nanoparticle concentrations are not directly comparable with those of dissolved Na_2_SeO_3_. In a separate study, dissolved sodium selenite was supplied through the hydroponic nutrient solution for 15 d [24]. By contrast, the present study used daily foliar Na_2_SeO_3_ pretreatment for 7 d, followed by 2 d of PEG-induced drought. Differences in Se formulation, application route, treatment frequency and duration, genotype, developmental stage and stress context may therefore account for the different effective and phytotoxic concentration ranges. Because tissue Se concentration and speciation were not measured, these nominal concentrations cannot be interpreted as equivalent internal Se exposure. Accordingly, 20 mg L^−1^ Na_2_SeO_3_ should be regarded as the most effective concentration among the three treatments tested under the present conditions, rather than as a universal optimum for *B. napus*.

The biochemical results in this study help explain the difference between the 20 and 40 mg L^−1^ Na_2_SeO_3_ treatments. At 20 mg L^−1^ Na_2_SeO_3_, foliar pretreatment reduced oxidative stress under drought without further increasing antioxidant enzyme activities. MDA, O_2_·^−^ and H_2_O_2_ moved toward the values in CK, while CAT, POD and SOD activities declined from the high levels observed under drought (Figure 2). This pattern is more consistent with improved redox homeostasis and lower oxidative pressure than with stronger enzymatic defence. At 40 mg L^−1^ Na_2_SeO_3_, the protective effect was weaker. At sufficiently high Se supply, Se can be assimilated through sulfur metabolic pathways into selenocysteine and selenomethionine, which may be nonspecifically incorporated into proteins in place of cysteine and methionine [37,38]. This process can disrupt protein function and increase oxidative pressure. Because Se accumulation and speciation were not measured, this mechanism remains a possible explanation for the reduced benefit at the higher treatment rather than direct evidence of phytotoxicity at 40 mg L^−1^ Na_2_SeO_3_.

These physiological and biochemical results justified selecting D+20 for the transcriptomic and metabolomic analyses. This allowed the molecular basis of the protective Se response to be examined under the most effective treatment condition.

### 3.2. Transcriptomic and Metabolomic Reprogramming Under Se-Mediated Drought Tolerance

The transcriptome and metabolome of CK, D and D+20 rapeseed seedlings were profiled to examine molecular changes associated with the protective Se response. Drought caused broad changes in gene expression in rapeseed seedlings. This agrees with previous studies showing large transcriptional responses in *B. napus* under PEG treatment or water deficit [39,40]. The smaller DEG set in D+20 vs. CK (Figure 3) was consistent with partial attenuation of the transcriptional changes caused by drought. However, D+20 vs. D still contained a large number of DEGs. Thus, Se did not simply return the seedlings exposed to drought to the control state. Instead, it produced a distinct transcriptional response under drought.

Separate enrichment of upregulated and downregulated genes clarified the transcriptomic response (Figure 4). In D vs. CK, terms and pathways related to photosynthesis were concentrated among downregulated genes, whereas hormone responses and carbohydrate metabolism were more prominent among upregulated genes. This pattern is consistent with reports that drought restricts photosynthesis and carbon assimilation [41]. In D+20 vs. D, photosynthesis, carbon fixation, porphyrin metabolism and related functions were concentrated among upregulated genes. This contrast suggests that Se partly countered drought-associated transcriptional changes in photosynthesis-related pathways. In D+20 vs. CK, hormone responses and chromatin remodelling were prominent among upregulated genes, whereas several secondary metabolic pathways were represented among downregulated genes.

The exploratory metabolomic analysis showed contrasting patterns in D vs. CK and D+20 vs. D. In D vs. CK, more candidate DAMs decreased than increased (Figure 5), suggesting that drought suppressed part of the metabolic profile. Similar transcriptomic and metabolomic changes have been reported in *B. napus* seedlings under water deficit [42,43]. In D+20 vs. D, more DAMs increased than decreased, suggesting a shift in the metabolite-feature profile after Se pretreatment. These features mapped to pathways including starch and sucrose metabolism, porphyrin metabolism, amino acid-related pathways, lipid-related pathways and selenocompound metabolism (Figure 6). These pathways are related to carbon use, chlorophyll metabolism, membrane adjustment and Se metabolism [44,45]. Together, these results link Se pretreatment to changes in gene expression and the metabolite-feature profile under drought. The qRT-PCR analysis of selected genes supported the RNA-seq trends (Figure S1). Pathways represented at both the gene and metabolite levels are discussed below.

### 3.3. Photosynthesis and Porphyrin Metabolism Were Associated with Lower Oxidative Pressure Under Se Pretreatment

Separate enrichment of upregulated and downregulated genes revealed a clear directional pattern. In D vs. CK, GO terms and KEGG pathways involving photosynthesis were concentrated among downregulated genes. In D+20 vs. D, pathways involving photosynthesis, carbon fixation and porphyrin metabolism were concentrated among upregulated genes (Figure 4). Integrated KEGG analysis also showed that porphyrin metabolism was represented at both the gene and metabolite-feature levels in all three comparisons (Figure 7), indicating that this pathway was responsive to both drought and Se pretreatment. In D+20 vs. D, its representation at both levels, together with the enrichment of photosynthesis-, carbon fixation-, and porphyrin-related pathways among upregulated genes, suggests that the Se response under drought involved photosynthesis and chlorophyll metabolism in addition to changes in antioxidant enzyme activity.

Drought can restrict CO_2_ assimilation and disturb chloroplast electron transport. When the photosynthetic electron transport chain becomes excessively reduced, electrons may be transferred to oxygen, leading to ROS formation in chloroplasts [9]. This directional pattern suggests that Se partly countered the effect of drought on genes involved in photosynthesis. Previous studies reported improved photosynthetic performance or chlorophyll content after suitable Se treatments in several crops [46,47,48]. Se nanoparticle treatment under drought also increased several chlorophyll precursors in *B. napus* [25]. The reductions in MDA content, O_2_·^−^ production rate and H_2_O_2_ content in D+20 relative to D were consistent with lower oxidative pressure.

Porphyrin metabolism provides another link between photosynthesis and oxidative stress. This pathway produces tetrapyrroles required for chlorophyll biosynthesis [49]. Excess or unbound intermediates such as protochlorophyllide can act as photosensitisers and generate ^1^O_2_ upon illumination [50]. Drought can suppress the chlorophyll branch of tetrapyrrole biosynthesis [51]. However, the consequences of such changes depend on whether the intermediates are efficiently converted into chlorophyll or accumulate in photosensitising forms. Pathways related to porphyrin and chlorophyll metabolism have also been reported to respond to drought in cotton [52]. Sustained porphyrin status was associated with drought tolerance and lower oxidative damage in transgenic rice [53].

In the present study, the candidate metabolite features mapped to porphyrin metabolism across the three pairwise comparisons included features putatively annotated as protochlorophyllide, pheophorbide A, and pheophytin A (Tables S5 and S7). In D+20 vs. D, the features annotated as protochlorophyllide and pheophytin A showed higher relative abundance and were accompanied by lower O_2_·^−^ production, H_2_O_2_ content, and MDA content. These parallel changes suggest that Se pretreatment was associated with remodelling of tetrapyrrole and chlorophyll metabolism. However, their functional significance cannot be determined from the present data because the metabolite identities were not confirmed with authentic standards, and chlorophyll content, pathway flux, and ^1^O_2_ production were not directly measured.

Many studies have linked the protective effect of Se under drought or other abiotic stresses to stronger antioxidant enzyme activity, including higher SOD, POD, CAT and APX activities [14,29,54,55]. In this study, however, CAT, POD and SOD activities declined from the high levels observed under drought, while the O_2_·^−^ production rate, H_2_O_2_ content and MDA content also decreased in D+20 (Figure 2). This pattern is more consistent with lower oxidative pressure than with stronger enzymatic scavenging. The difference from earlier reports may reflect variation in Se concentration, Se form, plant species, tissue type and sampling time. Thus, the combined results support the possible involvement of photosynthesis and porphyrin metabolism in the Se response under drought.

### 3.4. Phenylpropanoid and Selenocompound Metabolism Were Associated with Redox Responses Beyond Antioxidant Enzymes

Beyond the photosynthesis and porphyrin responses discussed above, the integrated analysis identified phenylpropanoid and selenocompound pathways. Phenylpropanoid biosynthesis was represented by both DEGs and candidate DAMs in the two comparisons involving D+20. In the separate KEGG analysis, however, this pathway was among the top-ranked pathways for downregulated genes in both D+20 vs. CK and D+20 vs. D. This pattern is consistent with pathway remodelling rather than general activation. Selenocompound metabolism was represented at both the gene and metabolite-feature levels only in D+20 vs. D (Figure 7, Table S7). Thus, the phenylpropanoid signal was present in both comparisons involving D+20, whereas the selenocompound signal was specific to D+20 vs. D.

Phenylpropanoid biosynthesis produces phenolic acids, flavonoids and lignin precursors. Phenolic compounds and flavonoids can contribute to ROS scavenging and stress protection [56,57]. Changes in phenylpropanoid metabolism after Se treatment have been reported in other species. In pak choi, Se altered key biosynthetic genes, including *PAL*, *4CL*, *CYP73A*, *F3H*, *FLS* and *DFR*, and increased flavonoid metabolites such as kaempferol and quercetin [58]. In melon, Se increased total phenols, flavonoids and PAL activity [59]. In the present study, O2PLS identified a metabolite feature with a putative match to [6]-gingerol among the high-loading features contributing to transcriptome-metabolome covariance in D+20 vs. D (Figure 8). This match is consistent with secondary metabolite remodelling, but it does not demonstrate increased phenylpropanoid production. A contribution to redox buffering remains possible and requires targeted validation.

The selenocompound signal in D+20 vs. D is relevant because Se was supplied as sodium selenite (Na_2_SeO_3_). Selenite metabolism intersects with sulfur metabolism, and absorbed selenite can be converted into organic Se compounds, including selenocysteine, selenomethionine and methylselenocysteine derivatives [37]. Studies in *B. napus* have also shown that inorganic Se can be converted into organic Se species such as selenocystine, methylselenocysteine and selenomethionine [26,60]. In D+20 vs. D, a candidate metabolite feature showed a putative match to Se-methylselenocysteine, together with several features bearing sulfur-containing annotations. GO analysis also placed sulfate transport and sulfur compound transport among the leading terms for downregulated genes (Figure 4). Together, these results suggest changes in sulfur-related transport and metabolite features after Na_2_SeO_3_ pretreatment, rather than uniform activation of sulfur metabolism. Glutathione and related thiols play important roles in plant redox balance and ROS detoxification [61,62]. However, GSH and GSSG were not among the candidate DAMs, and neither Se speciation nor glutathione pools were directly measured. These results should therefore be interpreted as pathway associations rather than direct evidence of organic Se formation or altered glutathione status.

Overall, the integrated results suggest that phenylpropanoid and selenocompound metabolism were associated with redox responses beyond antioxidant enzymes. The phenylpropanoid signal was consistent with pathway remodelling. The selenocompound signal and changes in processes related to sulfur were consistent with responses to the applied Na_2_SeO_3_. Together with photosynthesis and porphyrin metabolism, these pathways may be associated with the lower oxidative pressure observed in D+20. Because the metabolite annotations were based on automated processing and database matching and were not confirmed with authentic standards, these interpretations remain preliminary and require targeted validation.

## 4. Materials and Methods

### 4.1. Plant Material and Growth Conditions

Seeds of *Brassica napus* cv. Zhongshuang 11 (ZS11) were sterilised in 1% (*v*/*v*) NaClO (Sinopharm Chemical Reagent Co., Ltd., Shanghai, China) for 8 min and rinsed five times with deionised water. The seeds were placed on wet gauze and maintained in an incubator (RXZ-500D, Ningbo Jiangnan Instrument Factory, Ningbo, China) at approximately 65% relative humidity. The light intensity was 392–415 μmol m^−2^ s^−1^, with 16 h of light at 25 °C and 8 h of darkness at 18 °C. This photoperiod was used as the standard growth chamber regime rather than to meet a specific dark requirement for germination. Radicle emergence began approximately 3 d after sowing, and germination of the seed lot was essentially complete by day 7. Because some seedlings emerged earlier, the uniform seedlings selected on day 7 had generally developed two true leaves. Uniform 7-day-old seedlings were then transferred to half-strength Hoagland nutrient solution and grown under the same conditions until the four-leaf stage. The nutrient solution was completely renewed every 24 h with freshly prepared solution to maintain nutrient supply and root-zone oxygenation. At the four-leaf stage, a foliar pretreatment was applied prior to drought stress. Plants were sprayed daily for 7 consecutive days with either deionised water or varying concentrations of Se supplied as Na_2_SeO_3_ (Sinopharm Chemical Reagent Co., Ltd., Shanghai, China). Following this 7 d foliar application period, drought stress was imposed for 2 d by replacing the standard medium with 1/2 Hoagland nutrient solution containing 10% (*w*/*v*) PEG 6000 (Sinopharm Chemical Reagent Co., Ltd., Shanghai, China). The five treatment groups were designed as follows: CK, daily foliar spray of deionised water for 7 d, followed by 2 d in standard 1/2 Hoagland nutrient solution (control); D, daily foliar spray of deionised water for 7 d, followed by 2 d in 1/2 Hoagland nutrient solution containing 10% (*w*/*v*) PEG 6000; D+10, daily foliar spray of 10 mg L^−1^ Na_2_SeO_3_ for 7 d, followed by 2 d in 1/2 Hoagland nutrient solution containing 10% (*w*/*v*) PEG 6000; D+20, daily foliar spray of 20 mg L^−1^ Na_2_SeO_3_ for 7 d, followed by 2 d in Hoagland nutrient solution containing 10% (*w*/*v*) PEG 6000; D+40, daily foliar spray of 40 mg L^−1^ Na_2_SeO_3_ for 7 d, followed by 2 d in 1/2 Hoagland nutrient solution containing 10% (*w*/*v*) PEG 6000. Using the molecular mass of anhydrous Na_2_SeO_3_ (172.95 g mol^−1^), the 10, 20 and 40 mg L^−1^ Na_2_SeO_3_ solutions corresponded to 57.8, 115.6 and 231.3 μmol L^−1^ Na_2_SeO_3_, or elemental Se concentrations of 4.57, 9.13 and 18.26 mg Se L^−1^, respectively. Each treatment included three biological replicates. Following the 2 d drought treatment, seedlings were harvested individually, immediately frozen in liquid nitrogen, and stored at −80 °C until use for further analysis.

### 4.2. Stress Tolerance Assay

Following the 2 d drought treatment, representative seedlings were photographed against a matte background. Plants were harvested from the nutrient solution, rinsed briefly in deionised water, and blotted dry. Whole-plant fresh weight was measured to 0.001 g (ME204E, Mettler-Toledo GmbH, Greifensee, Switzerland), recorded per plant, and averaged within each biological replicate.

For biochemical analyses, 0.1 g frozen leaf tissue was directly used for extraction. The contents of MDA, H_2_O_2_, and O_2_·^−^ production rate, as well as the activities of SOD, CAT, and POD, were all measured using commercial assay kits (Suzhou Comin Biotechnology Co., Ltd., Suzhou, China) in accordance with the manufacturer’s instructions. All biochemical assays were run in triplicate technical replicates within each biological replicate.

### 4.3. Transcriptome Profiling

Based on the physiological results, D+20 was selected as the most effective Se treatment for transcriptome analysis. Three groups, CK, D and D+20, were included, with three biological replicates per group. RNA sequencing was performed by Novogene Co., Ltd. (Beijing, China). Total RNA was extracted from seedlings using the RNeasy Plant Mini Kit (Qiagen, Germany). RNA integrity was evaluated with an Agilent 2100 Bioanalyzer (Agilent Technologies, Santa Clara, CA, USA), and only samples with RIN values of at least 7.5 were used for library preparation. Strand-specific RNA-seq libraries were constructed using the NEBNext Ultra II Directional RNA Library Prep Kit (New England Biolabs, Ipswich, MA, USA). The libraries were sequenced on an Illumina NovaSeq 6000 platform to generate 150 bp paired-end reads, with at least 6 Gb of clean data obtained for each library. Raw reads were filtered using fastp v0.23 to remove adapters and low-quality sequences [63]. The clean reads were then mapped to the *B. napus* ZS11 reference genome using HISAT2 [64]. Gene read counts were calculated with featureCounts based on the ZS11 genome annotation. Differential expression analysis was performed using DESeq2 [65]. Genes with |log_2_FC| ≥ 1 and *p* < 0.05 were defined as differentially expressed genes (DEGs). GO and KEGG enrichment analyses were conducted using the hypergeometric test, followed by Benjamini–Hochberg correction for multiple testing. Terms and pathways with *p* < 0.05 were considered significantly enriched.

### 4.4. Metabolome Profiling

Based on the physiological results, D+20 was selected as the most effective Se treatment for metabolome analysis. Three groups, CK, D and D+20, were included, with three biological replicates per group. Untargeted LC–MS/MS metabolomic profiling was performed by Novogene Co., Ltd. (Beijing, China). Frozen leaf samples were ground into fine powder in liquid nitrogen, and approximately 80 mg fresh weight was extracted with 1.0 mL of precooled 70% (*v*/*v*) methanol containing internal standards. The mixture was vortexed, sonicated for 10 min at 4 °C, and centrifuged at 12,000× *g* for 15 min at 4 °C. The supernatant was collected for subsequent LC-MS/MS analysis. Pooled quality-control samples were prepared by mixing equal aliquots from all extracts and were used to monitor the stability of the analytical system [66].

Untargeted metabolomic profiling was performed using a Vanquish UHPLC system (Dionex Softron GmbH, part of Thermo Fisher Scientific, Germering, Germany) coupled to an Orbitrap Exploris 240 mass spectrometer (Thermo Fisher Scientific (Bremen) GmbH, Bremen, Germany). Chromatographic separation was achieved on a Hypersil Gold column (100 × 2.1 mm, 1.9 μm) at a flow rate of 0.2 mL min^−1^ using water containing 0.1% formic acid as mobile phase A and methanol as mobile phase B. The 12 min gradient was 2% B at 0–1.5 min, 2–85% B at 1.5–3.0 min, 85–100% B at 3.0–10.0 min, 100–2% B at 10.0–10.1 min, and 2% B at 10.1–12.0 min. Data were acquired in positive and negative electrospray-ionisation modes using a spray-voltage magnitude of 3.5 kV, a capillary temperature of 320 °C, sheath- and auxiliary-gas settings of 35 and 10, respectively, and an auxiliary-gas heater temperature of 350 °C. MS/MS spectra were acquired in data-dependent acquisition mode, in which each full-MS survey scan was followed by automatic selection and fragmentation of precursor ions meeting the predefined intensity criteria.

Raw LC-MS/MS files were converted to mzXML format using MSConvert in ProteoWizard and processed using XCMS (v3.1.3) for peak detection, retention-time correction, feature alignment and peak integration [67]. Putative metabolite annotations were assigned by matching adduct information, accurate mass within a 10 ppm tolerance and MS/MS spectra against the service provider’s in-house spectral database, NovoMetDB. Following the predefined preprocessing workflow, features with missing values in more than 50% of all non-QC samples included in the original preprocessing dataset were removed before imputation. This filtering step was used to exclude highly sparse features and limit reliance on extensively imputed values [68]. The remaining missing values were imputed using the k-nearest neighbours method [69]. Background ions were removed using blank samples, and integrated peak areas were normalised using a total peak-area-based procedure. Features with a coefficient of variation greater than 30% across the pooled QC samples were excluded. After these procedures, 2605 positive-mode and 1403 negative-mode features, comprising 4008 features in total, were retained for downstream analysis. The retained features were mapped to KEGG pathways for functional classification [70].

The normalised metabolite matrix was imported into SIMCA 14 for multivariate statistical analysis. Principal component analysis (PCA) was used to assess sample distribution and analytical reproducibility. Orthogonal partial least-squares discriminant analysis (OPLS-DA) was then performed for pairwise comparisons among CK, D and D+20. Candidate DAMs were screened using VIP ≥ 1.0, raw *p* < 0.05, and FC > 1.5 or FC < 0.67. To assess multiple testing, Benjamini–Hochberg correction was applied separately to the raw *p* values of all 4008 retained metabolite features in each pairwise comparison. The resulting candidate DAMs were used for exploratory KEGG pathway analysis and subsequent integration with the transcriptome data.

### 4.5. Multi-Omics Integration

Transcriptome and metabolome datasets were integrated at the pathway level through KEGG mapping and at the feature level through bidirectional orthogonal projection to latent structures (O2PLS). Only KEGG pathways containing at least one DEG and at least one candidate DAM were retained for cross-omics display.

### 4.6. qRT-PCR Validation of Selected Genes

To validate the RNA-seq results, representative DEGs were selected for quantitative real-time PCR (qRT-PCR) analysis in CK, D and D+20 seedlings. Total RNA was extracted from rapeseed leaf samples and reverse-transcribed into cDNA. Primers were designed based on the *B. napus* ZS11 reference sequences and are listed in Table S1. qRT-PCR was performed on an Applied Biosystems 7500 Real-Time PCR System (Applied Biosystems, Foster City, CA, USA) using a SYBR Premix Ex Taq Kit (Takara Bio Inc., Kusatsu, Shiga, Japan; Cat. No. DRR041A), with *18S rRNA* used as the internal reference gene. Melting-curve analysis was performed to confirm primer specificity. Relative transcript levels were calculated using the 2^−ΔΔCt^ method, with CK set to 1.0 for each gene. Three biological replicates were analysed, and the results are presented as mean ± SD.

### 4.7. Statistical Analysis

Physiological and biochemical data were expressed as the mean ± SD from three biological replicates. For biochemical assays, technical replicates were averaged within each biological replicate before statistical analysis. Treatment effects were tested by one-way analysis of variance (ANOVA), and mean separation was performed using Duncan’s multiple range test at *p* < 0.05 in IBM SPSS Statistics 26.0. Different lowercase letters in the figures denote significant differences among treatments. Graphs were prepared with OriginPro 2025.

## 5. Conclusions

This study explored the protective effect of foliar selenium in *Brassica napus* seedlings under drought stress simulated by PEG 6000. Selenium pretreatment improved seedling growth and reduced oxidative injury, with 20 mg L^−1^ Na_2_SeO_3_ showing the strongest effect among the tested concentrations. The physiological results indicate that selenium helped restore redox homeostasis mainly by lowering oxidative pressure, rather than by further activating antioxidant enzymes. Transcriptomic results, together with pathway-level patterns derived from putatively annotated metabolite features, suggest the possible involvement of photosynthetic regulation, porphyrin metabolism, phenylpropanoid biosynthesis and selenocompound metabolism. These pathway-level interpretations should be considered preliminary hypotheses requiring targeted validation.

## Data Availability

The RNA-seq raw reads generated in this study have been deposited in the NCBI Sequence Read Archive (SRA) under BioProject accession PRJNA1481826. The raw LC-MS/MS metabolomics data generated in this study have been deposited in OMIX at the National Genomics Data Center, China National Center for Bioinformation, under accession number OMIX018860.

## Supplementary Materials

The following supporting information can be downloaded at: https://www.mdpi.com/article/10.3390/ijms27167232/s1.

## Abbreviations

The following abbreviations are used in this manuscript:

PEG	polyethylene glycol
MDA	malondialdehyde
CAT	catalase
SOD	superoxide dismutase
ROS	reactive oxygen species
POD	peroxidase
CK	well-watered control
D	PEG-induced drought treatment without Na_2_SeO_3_ pretreatment
D+10/20/40	drought plus foliar pretreatment with 10/20/40 mg L^−1^ Na_2_SeO_3_
FW	fresh weight
DEG	differentially expressed gene
FC	fold change
GO	Gene Ontology
KEGG	Kyoto Encyclopedia of Genes and Genomes
LC–MS/MS	liquid chromatography–tandem mass spectrometry
PCA	principal component analysis
OPLS-DA	orthogonal partial least-squares discriminant analysis
QC	quality control
DAM	differentially accumulated metabolite feature
VIP	variable importance in projection
GSH	reduced glutathione
GSSG	oxidized glutathione

## References

[1] Cook B.I., Mankin J.S., Marvel K., Williams A.P., Smerdon J.E., Anchukaitis K.J. (2020). Twenty-first century drought projections in the CMIP6 forcing scenarios. Earth’s Future.

[2] Douville H., Allan R.P., Arias P.A., Betts R.A., Caretta M.A., Cherchi A., Mukherji A., Raghavan K., Renwick J. (2022). Water remains a blind spot in climate change policies. PLoS Water.

[3] Batool M., El-Badri A.M., Hassan M.U., Yang H.Y., Wang C.Y., Yan Z.K., Jie K., Wang B., Zhou G. (2023). Drought stress in *Brassica napus*: Effects, tolerance mechanisms, and management strategies. J. Plant Growth Regul..

[4] Secchi M.A., Fernandez J.A., Stamm M.J., Durrett T., Prasad P.V.V., Messina C.D., Ciampitti I.A. (2023). Effects of heat and drought on canola (*Brassica napus* L.) yield, oil, and protein: A meta-analysis. Field Crops Res..

[5] Zhi X.M., Bian X.H., Yu J.L., Xiao X.L., Duan B., Huang F.Y., Jiang Z., Zhou G., Ma N. (2024). Comparative metabolomics analysis of tolerant and sensitive genotypes of rapeseed (*Brassica napus* L.) seedlings under drought stress. Agric. Water Manag..

[6] Zandalinas S.I., Mittler R. (2022). Plant responses to multifactorial stress combination. New Phytol..

[7] Mittler R., Zandalinas S.I., Fichman Y., Van Breusegem F. (2022). Reactive oxygen species signalling in plant stress responses. Nat. Rev. Mol. Cell Biol..

[8] Alam P., Faizan M., Arif Y., Azzam M.M., Hayat S., Afzal S., Albalawi T. (2025). Reactive oxygen species: Balancing agents in plants. Front. Plant Sci..

[9] Foyer C.H., Hanke G. (2022). ROS production and signalling in chloroplasts: Cornerstones and evolving concepts. Plant J..

[10] Haider F.U., Liu T.H., Aguila L.C.R., Shahzad B., Habiba, Zhang P., Li X. (2026). Harnessing antioxidants for abiotic stress management: Mechanistic insights and prospects for sustainable agriculture. Antioxidants.

[11] Somagattu P., Chinnannan K., Yammanuru H., Reddy U.K., Nimmakayala P. (2024). Selenium dynamics in plants: Uptake, transport, toxicity, and sustainable management strategies. Sci. Total Environ..

[12] Shahidin, Wang Y., Wu Y.L., Chen T.X., Wu X.Y., Yuan W.J., Zhu Q., Wang X., Zi C. (2025). Selenium and selenoproteins: Mechanisms, health functions, and emerging applications. Molecules.

[13] Lanza M., dos Reis A.R. (2021). Roles of selenium in mineral plant nutrition: ROS scavenging responses against abiotic stresses. Plant Physiol. Biochem..

[14] Ahmad Z., Anjum S., Skalicky M., Waraich E.A., Tariq R.M.S., Ayub M.A., Hossain A., Hassan M.M., Brestic M., Sohidul Islam M. (2021). Selenium alleviates the adverse effect of drought in oilseed crops camelina (*Camelina sativa* L.) and canola (*Brassica napus* L.). Molecules.

[15] Tsivileva O. (2025). Selenium-containing nanoformulations capable of alleviating abiotic stress in plants. Int. J. Mol. Sci..

[16] Schiavon M., Pilon-Smits E.A.H. (2017). The fascinating facets of plant selenium accumulation: Biochemistry, physiology, evolution and ecology. New Phytol..

[17] Abbas S., Nidhi, Anjum N.A., Fatma M., Ahmad A. (2025). Assessment of the major biochemical and physiological mechanisms underlying green-synthesised selenium nanoparticles-mediated alleviation of lanthanum toxicity in mung bean (*Vigna radiata* L.). J. Hazard. Mater..

[18] Li W.M., Wang Y.L., Li J.J., Guo X.Y., Song Q.Q., Xu J. (2024). Selenite improves growth by modulating phytohormone pathways and reprogramming primary and secondary metabolism in tomato plants. Plant Physiol. Biochem..

[19] Liu C.L., Zhou G.J., Qin H.H., Guan Y.F., Wang T.Y., Ni W., Xie H., Xing Y., Tian G., Lyu M. (2024). Metabolomics combined with physiology and transcriptomics reveal key metabolic pathway responses in apple plants exposure to different selenium concentrations. J. Hazard. Mater..

[20] Huang F.Y., Li H.G., Chen L., Li T., Lian J.P., Dai W., Ma L., Zhou Y., Li Q., White J.C. (2026). Selenium supplementation alleviates drought-induced impacts in plants: A global meta-analysis. J. Agric. Food Chem..

[21] Pilon-Smits E.A.H. (2019). On the ecology of selenium accumulation in plants. Plants.

[22] White P.J. (2016). Selenium accumulation by plants. Ann. Bot..

[23] Prins C.N., Hantzis L.J., Quinn C.F., Pilon-Smits E.A.H. (2011). Effects of selenium accumulation on reproductive functions in *Brassica juncea* and *Stanleya pinnata*. J. Exp. Bot..

[24] Ulhassan Z., Gill R.A., Ali S., Mwamba T.M., Ali B., Wang J., Huang Q., Aziz R., Zhou W. (2019). Dual behavior of selenium: Insights into physio-biochemical, anatomical and molecular analyses of four *Brassica napus* cultivars. Chemosphere.

[25] Rezayian M., Niknam V. (2025). Modulatory effects of selenium nanoparticle against drought stress in canola plants. BMC Plant Biol..

[26] Zhan J.P., Qi M., Wang C., Wang X.F., Wang H.Z., Dun X.L. (2024). Precise determination of selenium forms and contents in selenium-enriched rapeseed seedlings and flowering stalks by HPLC-ICP-MS. J. Agric. Food Chem..

[27] Száková J., Praus L., Tremlová J., Kulhánek M., Tlustoš P. (2017). Efficiency of foliar selenium application on oilseed rape (*Brassica napus* L.) as influenced by rainfall and soil characteristics. Arch. Agron. Soil Sci..

[28] Liu X., Yang Y., Deng X., Li M., Zhang W., Zhao Z. (2017). Effects of sulfur and sulfate on selenium uptake and quality of seeds in rapeseed (*Brassica napus* L.) treated with selenite and selenate. Environ. Exp. Bot..

[29] Luo H.W., Xing P.P., Liu J.H., Pan S.G., Tang X.R., Duan M.Y. (2021). Selenium improved antioxidant response and photosynthesis in fragrant rice (*Oryza sativa* L.) seedlings during drought stress. Physiol. Mol. Biol. Plants.

[30] Hasanuzzaman M., Raihan M.R.H., Siddika A., Bardhan K., Hosen M.S., Prasad P.V.V. (2024). Selenium and its nanoparticles modulate the metabolism of reactive oxygen species and morpho-physiology of wheat (*Triticum aestivum* L.) to combat oxidative stress under water deficit conditions. BMC Plant Biol..

[31] El-Badri A.M., Hashem A.M., Batool M., Sherif A., Nishawy E., Ayaad M., Hassan H.M., Elrewainy I.M., Wang J., Kuai J. (2022). Comparative efficacy of bio-selenium nanoparticles and sodium selenite on morpho-physiochemical attributes under normal and salt stress conditions, besides selenium detoxification pathways in *Brassica napus* L.. J. Nanobiotechnol..

[32] Farooq M.A., Islam F., Ayyaz A., Chen W.Q., Noor Y., Hu W.Z., Hannan F., Zhou W. (2022). Mitigation effects of exogenous melatonin-selenium nanoparticles on arsenic-induced stress in *Brassica napus*. Environ. Pollut..

[33] Hasanuzzaman M., Fujita M. (2011). Selenium pretreatment upregulates the antioxidant defense and methylglyoxal detoxification system and confers enhanced tolerance to drought stress in rapeseed seedlings. Biol. Trace Elem. Res..

[34] Kong Y., Tian Y., Liu K., Li X., Yang Y., Feng Y., Rehman M., Fahad S., Maqbool Z., Malik L. (2025). Morphophysiological reconfiguration and antioxidant networking underpin selenium-mediated drought adaptation in *Nicotiana tabacum*. ACS Omega.

[35] Hasanuzzaman M., Bhuyan M., Raza A., Hawrylak-Nowak B., Matraszek-Gawron R., Al Mahmud J., Nahar K., Fujita M. (2020). Selenium in plants: Boon or bane?. Environ. Exp. Bot..

[36] Cunha M.L.O., Oliveira L.C.A., Silva V.M., Agathokleous E., Vicente E.F., dos Reis A.R. (2024). Selenium promotes hormesis in physiological, biochemical, and biological nitrogen fixation traits in cowpea plants. Plant Soil.

[37] Trippe R.C., Pilon-Smits E.A.H. (2021). Selenium transport and metabolism in plants: Phytoremediation and biofortification implications. J. Hazard. Mater..

[38] Hossain A., Skalicky M., Brestic M., Maitra S., Sarkar S., Ahmad Z., Vemuri H., Garai S., Mondal M., Bhatt R. (2021). Selenium biofortification: Roles, mechanisms, responses and prospects. Molecules.

[39] Prasad M., Shetty P., Pal A.K., Rigó G., Kant K., Zsigmond L., Nagy I., Shivaprasad P.V., Szabados L. (2025). Transcriptional and epigenomic changes in response to polyethylene glycol-triggered osmotic stress in *Brassica napus* L.. J. Exp. Bot..

[40] Wang J., Jiao J., Zhou M.J., Jin Z.Y., Yu Y.J., Liang M.X. (2019). Physiological and transcriptional responses of industrial rapeseed (*Brassica napus*) seedlings to drought and salinity stress. Int. J. Mol. Sci..

[41] Qiao M.Y., Hong C.H., Jiao Y.J., Hou S.J., Gao H.B. (2024). Impacts of drought on photosynthesis in major food crops and the related mechanisms of plant responses to drought. Plants.

[42] Xiong J.L., Ma N. (2022). Transcriptomic and metabolomic analyses reveal that fullerol improves drought tolerance in *Brassica napus* L. Int. J. Mol. Sci..

[43] Zhang R., Gong R., An Z., Li G., Dai C., Yi R., Dong J., Hu J. (2025). Integrated physiological, transcriptomic and metabolomic analyses of glossy mutant under drought stress in rapeseed (*Brassica napus* L.). Ind. Crops Prod..

[44] Fabregas N., Fernie A.R. (2019). The metabolic response to drought. J. Exp. Bot..

[45] White P.J. (2018). Selenium metabolism in plants. Biochim. Biophys. Acta Gen. Subj..

[46] Raza M.A.S., Aslam M.U., Valipour M., Iqbal R., Haider I., Mustafa A.E.Z.M., Elshikh M.S., Ali I., Roy R., Elshamly A.M. (2024). Seed priming with selenium improves growth and yield of quinoa plants suffering drought. Sci. Rep..

[47] Sita K., Sehgal A., Bhardwaj A., Bhandari K., Jha U., Prasad P.V.V., Singh S., Kumar S., Siddique K.H., Nayyar H. (2023). Selenium supplementation to lentil (*Lens culinaris* Medik.) under combined heat and drought stress improves photosynthetic ability, antioxidant systems, reproductive function and yield traits. Plant Soil.

[48] Ge X., Zhong Y.F., Zhang Y.X., Tang R.Y. (2026). Azole selenourea carboxylate enhances salt tolerance in rice through coordinated physiological and molecular responses. J. Agric. Food Chem..

[49] Wang P., Ji S.L., Grimm B. (2022). Post-translational regulation of metabolic checkpoints in plant tetrapyrrole biosynthesis. J. Exp. Bot..

[50] Lee K.P., Kim C. (2024). Photosynthetic ROS and retrograde signaling pathways. New Phytol..

[51] Karami S., Shiran B., Ravash R. (2025). Molecular investigation of how drought stress affects chlorophyll metabolism and photosynthesis in leaves of C3 and C4 plant species: A transcriptome meta-analysis. Heliyon.

[52] Han A., Fu W., Liusui Y., Zhong X., Zhang X., Wang Z., Li Y., Zhang J., Guo Y. (2025). Comparative transcriptome and metabolome profiling unveil genotype-specific strategies for drought tolerance in cotton. Front. Plant Sci..

[53] Phung T.H., Jung H.I., Park J.H., Kim J.G., Back K., Jung S. (2011). Porphyrin biosynthesis control under water stress: Sustained porphyrin status correlates with drought tolerance in transgenic rice. Plant Physiol..

[54] Saeed K., Hussain M.A., Abdalla M.A., Mühling K.H. (2025). Selenium increases the capacity of antioxidative defense and their accompanying metal cofactors in maize under sulfate salinity. Plant Stress.

[55] Jin L.L., Qiao Q., Yang L., Gao Y. (2025). Integrated physiological, transcriptomic, and metabolomic analyses reveal the response mechanisms of selenium (Se) and boron (B) under lead (Pb) stress in tobacco. Plant Cell Environ..

[56] Wang P.T., Liu W.C., Han C., Wang S.T., Bai M.Y., Song C.P. (2024). Reactive oxygen species: Multidimensional regulators of plant adaptation to abiotic stress and development. J. Integr. Plant Biol..

[57] Fu Y.H., Chen C., Pei P., Hao X.Y., Jin J.J., Shi S.J., Ge Q., Wang P., Li G., Fu G. (2025). The SnRK1 kinase TaSnRK1.10 positively regulates heavy metal chromium tolerance mainly by mediating reactive oxygen species homeostasis and activating the phenylpropanoid biosynthetic pathway in wheat. J. Hazard. Mater..

[58] Wang Y.Y., Sun P.H., Nie M.Y., Zhan J.Y., Huang L., Wu J.D., Zhang J., He X., Li N., Hu L. (2025). Integration of metabolomics and transcriptomics analyses reveals the effects of nano-selenium on pak choi. Sci. Rep..

[59] Kang L., Wu Y.L., Jia Y.J., Chen Z.D., Kang D.X., Zhang L., Pan C. (2023). Nano-selenium enhances melon resistance to *Podosphaera xanthii* by enhancing the antioxidant capacity and promoting alterations in the polyamine, phenylpropanoid and hormone signaling pathways. J. Nanobiotechnol..

[60] Ren L.J., Jiang W.G., Geng J.P., Yu T., Ji G.H., Zhang X.X., Wang X., Wang H. (2023). Sulfur levels regulate the absorption and utilization of selenite in rapeseed (*Brassica napus*). Environ. Exp. Bot..

[61] Dorion S., Ouellet J.C., Rivoal J. (2021). Glutathione metabolism in plants under stress: Beyond reactive oxygen species detoxification. Metabolites.

[62] Foyer C.H., Kunert K. (2024). The ascorbate-glutathione cycle coming of age. J. Exp. Bot..

[63] Chen S.F., Zhou Y.Q., Chen Y.R., Gu J. (2018). Fastp: An ultra-fast all-in-one FASTQ preprocessor. Bioinformatics.

[64] Kim D., Paggi J.M., Park C., Bennett C., Salzberg S.L. (2019). Graph-based genome alignment and genotyping with HISAT2 and HISAT-genotype. Nat. Biotechnol..

[65] Love M.I., Huber W., Anders S. (2014). Moderated estimation of fold change and dispersion for RNA-seq data with DESeq2. Genome Biol..

[66] Broeckling C.D., Beger R.D., Cheng L.L., Cumeras R., Cuthbertson D.J., Dasari S., Davis W.C., Dunn W.B., Evans A.M., Fernandez-Ochoa A. (2023). Current practices in LC-MS untargeted metabolomics: A scoping review on the use of pooled quality control samples. Anal. Chem..

[67] Domingo-Almenara X., Siuzdak G., Li S. (2020). Metabolomics data processing using XCMS. Computational Methods and Data Analysis for Metabolomics.

[68] Taylor S., Ponzini M., Wilson M., Kim K. (2022). Comparison of imputation and imputation-free methods for statistical analysis of mass spectrometry data with missing data. Brief. Bioinform..

[69] Do K.T., Wahl S., Raffler J., Molnos S., Laimighofer M., Adamski J., Suhre K., Strauch K., Peters A., Gieger C. (2018). Characterization of missing values in untargeted MS-based metabolomics data and evaluation of missing data handling strategies. Metabolomics.

[70] Kanehisa M., Furumichi M., Sato Y., Matsuura Y., Ishiguro-Watanabe M. (2025). KEGG: Biological systems database as a model of the real world. Nucleic Acids Res..

