# Supplementary material for: Integrated Physiological and Multi-Omics Analysis Reveals Selenium-Mediated Drought Tolerance in Brassica napus Seedlings
Source: Int J Mol Sci. 2026 Aug 13;27(16):7232. doi: 10.3390/ijms27167232 (PMC13513094; doi:10.3390/ijms27167232)
Supplement: Supplementary file 1 [file ijms-27-07232-s001.zip › Figure S2.pdf]

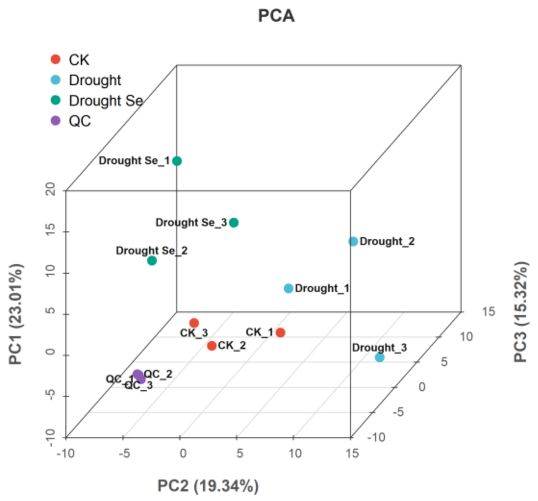

Figure S2. Principal component analysis (PCA) of the three treatment groups and pooled quality control (QC) samples. The close clustering of the QC samples supports the analytical stability and reproducibility of the LC–MS/MS dataset. CK, well-watered control; D, drought without selenium; D+20, drought plus foliar Na_2SeO_3 at 20 mg L^{-1}.
