# Supplementary material for: Integrated Physiological and Multi-Omics Analysis Reveals Selenium-Mediated Drought Tolerance in Brassica napus Seedlings
Source: Int J Mol Sci. 2026 Aug 13;27(16):7232. doi: 10.3390/ijms27167232 (PMC13513094; doi:10.3390/ijms27167232)
Supplement: Supplementary file 1 [file ijms-27-07232-s001.zip › Figure S1.pdf]

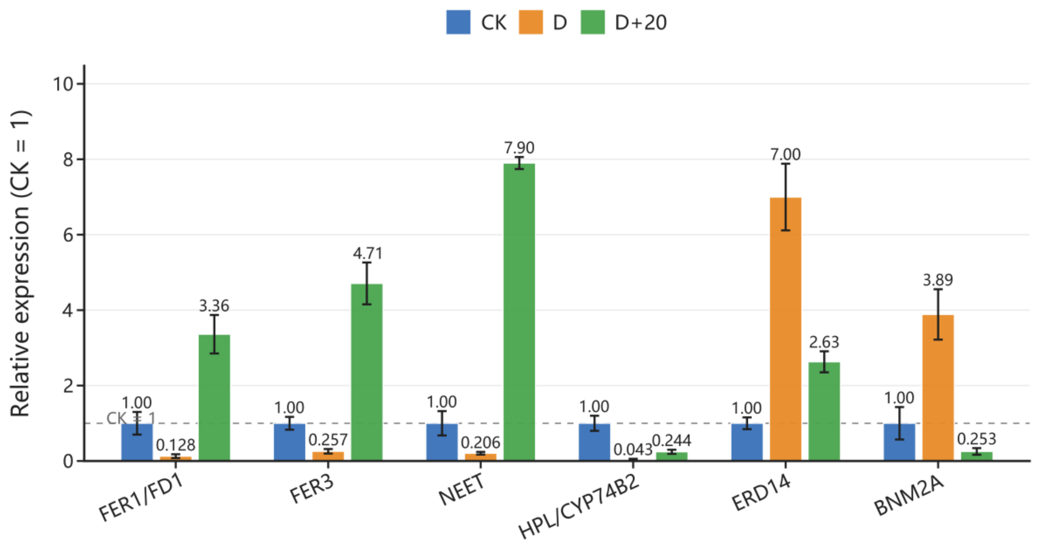

Figure S1. qRT-PCR validation of selected selenium-responsive genes in rapeseed leaves. Relative transcript levels of six selected genes, including *FER1/FD1*, *FER3*, *NEET*, *HPL/CYP74B2*, *ERD14* and *BNM2A*, were determined in CK, D and D+20 seedlings. CK represents the well-watered control, D represents 10% PEG 6000 induced drought stress, and D+20 represents seedlings pretreated with foliar selenium at 20 mg L^{-1} for 7 d followed by 10% PEG 6000 treatment. Expression levels were normalized to the internal reference gene *18S rRNA* and calculated using the $2^{-\Delta\Delta Ct}$ method, with CK set to 1.0 for each gene. Bars represent mean ± SD of three biological replicates. Numbers above the bars indicate mean relative expression values.
